# Endocrinological and inflammatory markers in individuals with spinal cord injury: A systematic review and meta-analysis

**DOI:** 10.1007/s11154-022-09742-9

**Published:** 2022-08-18

**Authors:** Gabriela Boehl, Peter Francis Raguindin, Ezra Valido, Alessandro Bertolo, Oche Adam Itodo, Beatrice Minder, Patricia Lampart, Anke Scheel-Sailer, Alexander Leichtle, Marija Glisic, Jivko Stoyanov

**Affiliations:** 1 Swiss Paraplegic Research, Nottwil, Switzerland; 2 Institute of Social and Preventive Medicine, University of Bern, Bern, Switzerland; 3 Graduate School for Health Sciences, University of Bern, Bern, Switzerland; 4 Department of Health Sciences and Medicine, University of Lucerne, Lucerne, Switzerland; 5 Department of Orthopedic Surgery, University of Bern, Bern Inselspital, Bern, Switzerland; 6 Public Health & Primary Care Library, University Library of Bern, University of Bern, Bern, Switzerland; 7 Swiss Paraplegic Centre, Nottwil, Switzerland; 8 University Institute of Clinical Chemistry, Inselspital, Bern University Hospital and University of Bern, Bern, Switzerland

**Keywords:** Spinal cord injury, Hormones, Growth factors, Metabolism, Inflammatory markers, Vitamin D

## Abstract

Spinal cord injury (SCI) can lead to dramatic physiological changes which can be a factor in developing secondary health conditions and might be reflected in biomarker changes in this elevated risk group. We focused specifically on the endocrine and inflammation profile differences between SCI and able-bodied individuals (ABI). Our aim was to determine the differences in inflammatory markers and endocrine profiles between SCI and ABI. We systematically searched 4 electronic databases for relevant studies. Human observational (cross-sectional, cohort, case–control) studies that compared biomarkers of interest between SCI and ABI population were included. Weighted mean difference between SCI and ABI was calculated using random-effects models. Heterogeneity was computed using I^2^ statistic and chi-squared test. Study quality was evaluated through the Newcastle–Ottawa Scale. The search strategy yielded a total of 2,603 studies from which 256 articles were selected for full-text assessment. Sixty-two studies were included in the meta-analysis. SCI individuals had higher levels of pro-inflammatory C-reactive protein and IL-6 than ABI. Creatinine and 25-hydroxyvitamin D_3_ levels were lower in SCI than ABI. Total testosterone levels and IGF-1 were also found to be lower, while cortisol and leptin levels were higher in SCI when compared to ABI. Accordingly, meta-regression, subgroup analysis, and leave-one-out analysis were performed, however, they were only able to partially explain the high levels of heterogeneity. Individuals with SCI show higher levels of inflammatory markers and present significant endocrinological changes when compared to ABI. Moreover, higher incidence of obesity, diabetes, osteoporosis, and hypogonadism in SCI individuals, together with decreased creatinine levels reflect some of the readily measurable aspects of the phenotype changes in the SCI group. These findings need to be considered in anticipating medically related complications and personalizing SCI medical care.

**Supplementary Information:**

The online version contains supplementary material available at 10.1007/s11154-022-09742-9.

## Introduction

Spinal cord injury (SCI) refers to any traumatic or non-traumatic damage to the spinal cord that leads to motor, sensory, and/or autonomic impairments below the affected area [1]. SCI specifically compromises the central nervous system (CNS), vasculature and blood–spinal cord barrier [2]. Worldwide, the prevalence of SCI is estimated from 236 to 4,187 per million inhabitants, with a growing incidence rate of 133 to 226 thousand cases of traumatic SCI per year [3]. Although the incidence is relatively low compared to other diseases, there is a tremendous economic burden for the healthcare system due to the high prevalence of secondary health conditions and the need for chronic care [4, 5]. Overall, SCI individuals have increased mortality and morbidity rates when compared to age-and sex- matched able-bodied individuals (ABI) [6].

Furthermore, the injury confers significant physiological changes that could lead to the development of various health conditions [7–9]. Individuals with SCI have a higher incidence of developing cardiovascular diseases (CVDs), pressure ulcers, neurogenic bladder, recurrent urinary tract, skin, and respiratory infections, as well as, several metabolic disorders [7, 8, 10]. SCI individuals are characterized by a constant low-grade chronic inflammatory state, having a main effect in post-SCI complications [11]. A prolonged inflammatory state leads to the release of proinflammatory cytokines which alter the functionality of barriers, tissues and organs, and consequently exposing the body to unfavorable conditions [12]. Low-grade chronic inflammation therefore, has a significant effect on neurodegeneration and functional recovery after SCI [13]. Moreover, circulating levels of inflammation markers such as C-reactive protein (CRP) and interleukin-6 (IL-6) have been shown to be significantly increased in individuals with chronic SCI, regardless of the duration and level of injury, when compared to ABI [11].

In addition, the loss of neurologic control from the injury leads to muscle atrophy and bone loss. This results in limitations on mobility and functioning of the individual that has ramifications on the development of other chronic diseases. Furthermore, the SCI population undergoes premature aging and accelerated deterioration of body systems that are also manifested by endocrine and inflammatory profile differences when compared to ABI [14–16]. SCI individuals not only experience a decline of several anabolic and somatotropic hormones -especially sex hormones- [18], but also have immune dysfunction [19]. Suppressed function of natural killer cells, neutrophils, macrophages and lymphocytes has been evidenced after SCI [20].

Several small studies have been conducted to characterize the inflammatory and endocrine profile of individuals with SCI [21–26]. These studies show varying effect estimates due to small study sizes and different comparison groups. In addition, some studies also show no significant differences among SCI and ABI individuals or present inconsistent findings amongst each other. Although there are a number of studies on hormones and inflammatory profiles in individuals with SCI [21–26], there have been no efforts to appraise the literature and synthesize the data. Thus, we reviewed the literature to determine the differences in inflammatory markers, hormones, and other related metabolites in individuals with SCI compared to ABI. Furthermore, we discussed the gaps in the literature to guide future research on biomarkers for health screening and promotion in SCI and ultimately provide basis to optimize medical care in this group.

## Methods

This is a systematic review and meta-analysis of observational studies that reported various inflammatory markers, hormones, and other metabolites levels between SCI and ABI.

### Data sources and search strategy

We conducted the review following a recently published guideline for systematic reviews and meta-analysis [27] and the Preferred Reporting Items for Systematic Reviews and Meta-analysis (PRISMA) statement [28]. The electronic search was performed using the following databases: MEDLINE (Ovid), EMBASE, Cochrane CENTRAL, and PubMed from inception until September 21, 2020 (date of the last search). In addition, the first 100 hits in Google Scholar were included to further the scope for eligible studies. The detailed search strategy was developed by medical information specialists following standard blood panel recommendations [29] and can be found in the Supplementary Information (SI) [30]. Studies comparing the following outcomes between SCI and ABI were included, (a) inflammatory markers (C-reactive protein, CRP; high sensitivity CRP, hsCRP; interleukin-6, IL-6; tumor necrosis factor-alpha, TNF-alpha), (b) insulin, (c) creatinine, (d) vitamin D [hydroxy -25(OH); dihydroxy 1,25 (OH)], (e) fasting glucose and (f) hormones and growth factors (total testosterone; free testosterone; thyroid-stimulating hormone, TSH; triiodothyronine, T3; luteinizing hormone, LH; follicle-stimulating hormone, FSH; growth hormone, GH; cortisol; adrenocorticotropic hormone, ACTH; adiponectin; aldosterone; insulin growth-like factor 1, IGF1; leptin; prolactin; parathyroid hormone, PTH; sex-hormone-binding globulin, SHBG). The full review protocol can be accessed online (International Prospective Register of Systematic Reviews, PROSPERO No. CRD42020210685).

### Study selection, eligibility criteria, and data extraction

We included observational studies (i.e., cross-sectional, cohort, case–control studies) on adults that compared inflammatory and endocrine levels markers between SCI and ABI. We excluded clinical trials, mechanistic studies, experiments on cells (*in-vitro* models), and animal studies (*in-vivo* models). Reviews, conference abstracts, cost-effectiveness, economic assessments, letters to editors, commentaries, and other non-peer-reviewed articles were excluded. No date and language restrictions were applied. Titles and abstracts were screened by at least two independent reviewers (GB/PFR and EV/AB/GPF/OAI). Full-text articles were extracted and reviewed for eligibility by two authors (GB and PFR). Adjudication was done by a third author (MG/JS) if consensus between the two reviewers could not be reached. Data extraction was performed separately by the two reviewers using a previously established template.

### Quality of evidence assessment

Study quality and risk of bias were assessed following the Newcastle–Ottawa Scale for cross-sectional, case–control, and cohort studies by the two reviewers (GB and PFR) [31]. In summary, this scale is based on three categories: (a) study selection (i.e., sample size, representativeness of the sample, and ascertainment of exposure), (b) comparability (i.e., factors that were compared between the groups other than the outcome), and (c) outcome (assessment and the statistical test used). A rating was given per category for each study with a maximum total score of 10. Studies with scores of 8–10 were classified as high quality, 5–7 as moderate quality, and 1–4 as low quality [27].

### Data synthesis and analysis

Biomarkers-of-interest levels, including mean and standard deviation (or standard error of the mean), median and interquartile range (or minimum and maximum values) were extracted. For data reported as medians, ranges, or 95% confidence interval; the means and standard deviations were calculated using previously defined methods [32]. Only baseline values were used in studies with repeated measures. For outcomes that were reported in different units, we converted the values into the International System of Units (SI) [33]. We computed for pooled means and standard deviation of SCI and ABI groups. Weighted mean difference (WMD) between SCI and ABI biomarker values was computed using the random-effects model by DerSimonian and Laird method [34]. A positive WMD signified that the pooled mean of individuals with SCI is higher than in ABI. A negative value meant that individuals with SCI have lower pooled means compared to ABI.

Study characteristics (i.e., location of the study, number of participants, source of control population, matching variables), participant characteristics (i.e., age, sex, health status), and injury characteristics (i.e., duration of injury, injury completeness, injury level) were collected from each study. Heterogeneity was evaluated using the Cochran's squared test (x^2^) and Higgins I^2^ statistic test. The level of heterogeneity was classified as low (I^2^ ≤ 25%), moderate (I^2^ > 25%, < 75%), or high (I^2^ ≥ 75%) [35]. To further explore heterogeneity, we did stratified analyses using study, participant, and injury characteristics as strata. We also used random-effects meta-regression for study, participant, and injury characteristics with continuous data. Both stratified analysis and meta-regression were conducted for outcomes with ten or more studies. A leave-one-out analysis was also performed to evaluate if a single study affects the overall weighted mean difference. This analysis iteratively removes one study at a time and recomputes the weighted mean difference to detect any notable variation. Publication bias was explored through funnel plots and Egger's test for outcomes with more than eight studies [36]. STATA 16.1 (Stata Corporation, College Station, Texas) was used for statistical analysis and p-values < 0.05 were considered statistically significant.

## Results

### Literature search and study characteristics

**Fig. 1:**
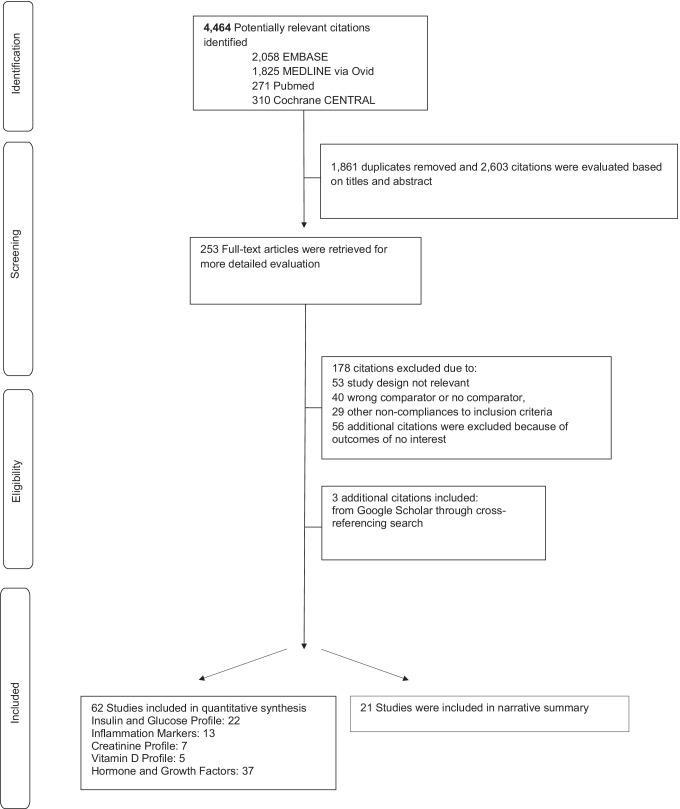
Flow chart of studies included in the review

The search strategy yielded 4,464 records, from which 1,861 were removed as duplicates. We screened 2,603 titles and abstracts, from which 256 articles were selected for full-text assessment, Fig. 1. Three additional citations were manually searched and included in the pool of included studies. Overall, 62 studies provided information on targeted outcomes in both ABI and SCI individuals and were included in the meta-analysis. A detailed summary of the studies included in the meta-analysis can be found in Table S1 [30], and the reason for the exclusion of other studies can be found in Table S2 [30].

Study characteristics of all studies included in the meta-analysis can be found in Table 1. Most of the studies were conducted among males (42/62, 68%), had a sample size below 100 participants (46/62, 74%), included both individuals with tetraplegia and paraplegia (48/62, 77%), and were conducted in North America (25/62, 40%). The mean age ranged from 16 to 64 years and the mean injury duration ranged from < 1 to 29 years. One-third of the studies (19/62, 31%) included complete injury, 40% (25/62) studies had both complete and incomplete injury, while 29% (18/62) of studies had no information. Most of the studies were classified by the Newcastle–Ottawa scale as moderate quality (44/62, 71%), while the rest of the studies were classified as good quality (18/62, 29%) Table 1.

**Table 1:** Characteristics of studies included in the meta-analysis (n = 62)

**Characteristics**	**No. of studies**	**References**
Level of injury		
Tetraplegia	6	[24, 85, 92, 97, 108, 132]
Paraplegia	4	[95, 101, 118, 124]
Mixed	48	[11, 22, 26, 37, 47, 56, 57, 60, 65–67, 84, 86–91, 94, 93, 96, 99, 100, 102–107, 109–115, 117, 119–122, 125–127, 129–131, 133]
Not reported	4	[43, 98, 128, 135]
Proportion of complete injury		
100%	19	[11, 65, 66, 85, 88, 92, 97, 98, 108, 111, 112, 118, 119, 122, 127, 129–132]
Mixed	25	[22, 26, 47, 84, 86, 87, 90, 91, 94, 99, 100, 102–106, 109, 113, 115, 117, 121, 124–126, 133, 135]
Not reported	18	[24, 37, 43, 56, 57, 60, 67, 89, 93, 95, 96, 99, 101, 107, 110, 114, 120, 128]
Duration of injury (years), range		
≤ 1–10	21	[26, 60, 65, 84, 86, 88, 90, 96, 104, 105, 107, 109, 111, 118, 119, 122, 126, 127, 129, 130, 132]
> 10	26	[11, 22, 24, 37, 47, 56, 66, 67, 85, 87, 89, 91, 94, 99, 100, 102, 103, 106, 110, 113, 117, 122, 124, 125, 131, 135]
Not reported	15	[43, 57, 95, 97, 98, 101, 108, 112, 114, 115, 120, 121, 128]
Sex		
Male only	42	[11, 22, 26, 43, 47, 57, 60, 65–67, 85, 86, 88, 92, 94, 96–99, 103, 106–115, 117–122, 129–132, 135]
Both	16	[24, 37, 84, 87, 89–91, 95, 101, 102, 104, 124–127, 133]
Female only	1	[100]
Not reported	3	[56, 105, 128]
Study size		
< 50	29	[24, 37, 43, 66, 84, 85, 87–89, 94, 95, 97–100, 110, 111, 114, 115, 117, 118, 120, 121, 126–130, 132]
50–100	17	[26, 57, 65, 67, 86, 91, 92, 101, 105, 107–109, 113, 119, 122, 124, 125]
> 100	16	[11, 22, 47, 56, 60, 90, 93, 96, 102–104, 106, 112, 131, 133, 135]
Age, (years) range		
16–40	42	[11, 26, 43, 47, 65, 84–89, 91, 92, 94–99, 101–105, 107–111, 114, 115, 118, 119, 121, 124, 126, 127, 131–133, 135]
> 40–65	16	[24, 37, 56, 60, 66, 67, 90, 93, 100, 106, 112, 113, 117, 122, 125, 129]
Not reported	4	[57, 120, 128, 130]
Location		
Europe	12	[84, 85, 91, 97, 109, 110, 112, 113, 115, 118, 126, 130]
North America	25	[24, 37, 56, 57, 60, 66, 67, 87, 89, 90, 93, 95, 96, 98, 100, 101, 103, 114, 117, 120, 121, 124, 125, 127, 129]
South America	4	[86, 88, 107, 111]
Asia	19	[11, 22, 26, 43, 47, 65, 92, 102, 104–106, 108, 119, 122, 128, 131–133, 135]
Oceania	2	[94, 99]
Gender matching		
Yes	36	[11, 22, 26, 56, 65, 66, 87, 89, 90, 93, 94, 96–100, 102–106, 110–115, 117–122, 125, 127, 131]
No	26	[24, 37, 43, 47, 57, 60, 67, 84–86, 88, 91, 92, 95, 101, 107–109, 113, 124, 126, 128, 129, 132, 133, 135]
Age matching		
Yes	39	[11, 22, 24, 26, 65, 66, 87, 89–91, 93–100, 102–106, 110–115, 117–122, 125, 130, 131]
No	23	[37, 43, 47, 57, 60, 67, 84–86, 88, 92, 101, 107–109, 113, 124, 126, 128, 129, 132, 133, 135]
Health Status		
Healthy	54	[11, 22, 24, 26, 37, 47, 56, 57, 60, 65–67, 84–89, 91–94, 96–102, 104, 105, 107, 111–115, 117–122, 124, 125, 127–132]
Non- healthy	3	[43, 109, 126]
Mixed	3	[103, 106, 110]
Not provided	2	[90, 95]
Outcomes		
*Biomarkers Outcomes*		
• Insulin and Glucose Profile	22	[11, 86–90, 93–108]
• Inflammation Profile	13	[11, 24, 37, 84–91, 103, 108]
• Creatinine Profile	7	[11, 57, 84, 108–111]
• Vitamin D Profile	5	[56, 57, 60, 84, 111]
• Hormone Profile	37	[22, 26, 43, 47, 56, 65–67, 84, 87, 89, 94, 97, 98, 110–115, 117–122, 124–133, 135]
Study quality		
Moderate (5–7)	44	[11, 22, 24, 43, 47, 56, 57, 60, 65, 67, 86, 88, 89, 93, 95, 97–99, 101, 106–111, 113–115, 117, 119–122, 126–135]
Good (8–10)	18	[26, 37, 66, 84, 85, 87, 90, 94, 96, 100, 102–105, 112, 118, 124, 125]

### Inflammatory markers

Based on the findings from 13 studies on inflammation-related markers including 532 SCI and 437 ABI individuals, we found that IL-6 (WMD 2.52 pg/mL, 95% confidence interval (CI) 1.82, 3.21), I^2^ 81.1%, p^χ2^ 0.001) and CRP (WMD 2.79 mg/L, 95% CI 1.75, 3.83, I^2^ 87.3%, p^χ2^ < 0.001) were significantly higher in the SCI group than the ABI group. No significant differences were found between the two groups regarding hsCRP and TNF-α.

### Creatinine and vitamin D

From the seven studies comprising 260 SCI and 136 ABI, creatinine was found to be significantly lower (WMD -14.23 µmol/L, 95% CI -21.57, -6.89, I^2^ 90.3%, p^χ2^ < 0.001) in the SCI group compared to ABI. Five studies with 289 SCI and 123 ABI individuals compared vitamin D profiles between SCI and ABI populations. The 25-hydroxyvitamin D_3_ [25(OH)D] was significantly lower (WMD -10.32 nmol/L, 95%CI -20.47, -0.18, I^2^ 57.2%, p^χ2^ 0.053) in SCI individuals compared to ABI. No significant difference was found in 1,25-dihydroxyvitamin D [1, 25(OH)D] levels.

### Insulin and glucose

Twenty-two studies compared glucose and insulin profiles (1,073 SCI and 1,003 ABI), however, no significant differences between the two groups were found either in glucose (WMD -0.08 mmol/L, 95% CI -0.19, 0.03, I^2^ 83.8%, p^χ2^ < 0.001) or insulin (WMD 3.99 pmol/L, CI -2.84, 10.83, I^2^ 50.5%, p^χ2^ 0.019).

### Hormones and growth factors

Thirty-seven studies explored hormone and growth factors profiles between 1,149 SCI and 918 ABI. Results from the meta-analysis showed significantly lower total testosterone (WMD -2.61 nmol/L, 95% CI -4.42, -0.79, I^2^ 89.6%, p^χ2^ < 0.001) for the SCI group than ABI, however free testosterone (WMD -0.01 nmol/L, 95%CI -0.024, 0.004, I^2^ 81.8%, p < 0.001) was not found to be significantly different. Decreased levels were also found for IGF-1 (WMD -6.82 nmol/L, 95% CI -9.24,-4.40, I^2^ 0%, p^χ2^ 0.529). On the other hand, cortisol levels (WMD 103.43 nmol/L, 95%CI 10.75, 196.11, I^2^ 67.5%, p^χ2^ 0.026) and leptin levels (WMD 0.19 nmol/L, 95%CI 0.10, 0.27, I^2^ 53.1%, p^χ2^ 0.047) were shown to be higher in SCI individuals when compared to ABI. No significant differences were found in TSH, T3, LH, FSH, GH, ACTH, adiponectin, aldosterone, prolactin, PTH, and SHBG.

### Heterogeneity analysis, meta-regression, subgroup analysis and sensitivity analysis

Significant study heterogeneity (I^2^ > 75% and Cochran x^2^ p < 0.05) was found in weighted mean differences for CRP, IL-6, TNF-α, creatinine, 1, 25(OH)D, total testosterone, T3, LH, FSH, prolactin, PTH and SHBG (Table 2). We further performed subgroup analysis using study design, injury, and participant characteristics as strata (Table S3−S7) [30]. Time since injury and age-sex matching in the meta-analysis of FSH (Table S5) [30] and location and health status in the meta-analysis of insulin (Table S6) [30] were identified as potential sources of heterogeneity. We were not able to explain other sources of heterogeneity in cases where I^2^ was higher than 75% (Tables S3–S9) [30]. In meta-regression, number of participants was identified as another potential source of high heterogeneity in total testosterone, LH, and FSH. Larger studies tended to have lower weighted mean difference (β -0.040, 95% CI -0.075, -0.005), FSH (β -0.064, 95% CI-0.100,-0.027), and LH (β-0.064, 95% CI -0.100, -0.027) in SCI compared to ABI (Table S9 and Fig. S3) (17). Leave-one-out sensitivity analysis showed that our effect estimates were stable upon iteratively removing one study at a time. No single study influenced our overall estimates (Fig. S2) [30].

**Table 2:** Weighted mean difference of biomarkers among spinal cord injury and able-bodied population

**Outcome (units)**	**Studies which reported higher levels in SCI**	**Studies which reported lower levels in SCI**	**No association**	**Number of studies**	**SCI, N**	**ABI, N**	**Weighted Mean Difference**	**95% confidence interval**	**I**^**2**^ **test for heterogeneity**	**x**^**2**^ **test for heterogeneity** **(p**^**χ2**^**)**
**Inflammatory markers**										
CRP (mg/L)	[11, 23, 37, 84–86]	-	[87, 88]	8	330	249	2.79	1.75, 3.83*	87.3%	< 0.001*
hsCRP (mg/L)	[89]	-	[90]	2	114	113	0.07	-0.07, 0.20	66.3%	0.085
IL-6 (pg/mL)	[11, 91, 92]	-	[24]	4	150	104	2.52	1.82, 3.21*	81.1%	0.001*
TNF alpha (pg/mL)	[91]	-	[24]	2	68	45	18.51	-26.14, 63.15	85.0%	0.010*
**Insulin**										
Insulin (pmol/L)	[93, 94]	[95]	[11, 87, 89, 90, 96–101]	13	493	485	3.99	-2.84, 10.83	50.5%	0.019*
Glucose (mmol/L)	[102]	[93, 96, 103]	[11, 86–90, 94, 95, 97–101, 104–108]	22	1073	1003	-0.08	-0.19, 0.03	83.8%	< 0.001*
**Creatinine**										
Creatinine(µmol/L)	-	[11, 109–111]	[57, 84, 108]	7	260	136	-14.23	-21.57, -6.89*	90.3%	< 0.001*
**Vitamin D**										
25(OH)D (nmol/L)	-	[60, 84]	[56, 57, 111]	5	289	123	-10.32	-20.47, -0.18*	57.2%	0.053
1,25(OH)D (pmol/L)	[56]	-	[57]	2	140	64	6.82	-50.10, 63.73	96.8%	< 0.001*
**Hormone and Growth Factors**										
Total Testosterone (nmol/L)	-	[22, 47, 111–117]	[26, 65, 66, 110, 119–122]	18	601	512	-2.61	-4.42, -0.79*	89.6%	< 0.001*
Free Testosterone (nmol/L)	-	[25, 115, 117]	[26, 66, 123]	6	141	123	-0.01	-0.024, 0.004	81.8%	< 0.001*
TSH (mU/L)	-	-	[62, 65, 124]	3	135	99	-0.03	-0.26, 0.20	0.0%	0.490
T3 (nmol/L)	-	[125]	[65, 124, 126]	4	108	100	-0.05	-0.29, 0.19	82.4%	0.001*
LH (IU/L)	[26]	[22, 47]	[65, 66, 110, 111, 114, 116, 117, 119–123, 126]	15	452	365	0.27	-0.61, 1.14	85.6%	< 0.001*
FSH (IU/L)	[65, 110, 111, 121]	[22, 47]	[66, 114–117, 119, 123]	13	388	305	0.59	-1.24, 2.43	95.7%	< 0.001*
GH (µg/L)	-	-	[65, 66, 98]	3	48	42	-0.32	-0.67, 0.04	0.0%	0.550
Cortisol (nmol/L)	[43, 127]	-	[65, 126]	4	90	76	103.43	10.75, 196.11*	67.5%	0.026*
ACTH (pmol/L)	-	-	[126, 127]	2	36	28	1.59	-0.40, 3.58	0.0%	0.786
Adiponectin (µg/mL)	-	-	[87, 89]	2	29	29	0.92	-3.12, 4.96	24.6%	0.249
Aldosterone (pmol/l)	-	-	[128, 129]	2	32	22	100.62	-69.57, 270.81	40.2%	0.196
IGF-1 (nmol/L)	-	[65]	[66, 84, 97]	4	76	59	-6.82	-9.24, -4.40*	0.0%	0.529
Leptin (nmol/L)	[67, 89, 94, 97, 130, 131]	-	[98]	7	176	141	0.19	0.10, 0.27*	53.1%	0.047*
Prolactin (µg/L)	-	-	[22, 47, 65, 121, 126, 127]	6	209	134	0.80	-1.01, 2.61	76.5%	0.001*
PTH (ng/L)	-	-	[56, 62, 84]	3	203	104	4.17	-5.38, 13.72	87.2%	< 0.001*
SHBG (nmol/L)	-	[110]	[66, 111, 113, 132]	5	95	84	-0.85	-8.48, 6.79	78.1%	0.001*

A positive WMD signifies that the pooled means of individuals with SCI is higher than ABI. A negative value means that individuals with SCI has lower pooled means compared to ABI

*ABI* able-bodied individuals, *ACTH* adrenocorticotropic hormone, *CRP* c-reactive protein, *FSH* follicle stimulating hormone, *GH* growth hormone, *hsCRP* highly sensitive c-reactive protein, *IGF-1* insulin-like growth factor-1, *IL-6* interleukin 6, *LH* luteinizing hormone, *PTH* parathyroid hormone, *SCI* individuals with spinal cord injury, *SHBG* sex steroid-binding globulin, *TNF alpha* tumor necrosis factor alpha, *TSH* thyroid stimulating hormone, *T3* triiodothryoxine

^*^Indicates statistically significant result, p-value < 0.05

### Study quality and publication bias

Publication bias was explored for total testosterone, LH, FSH, CRP, and insulin Fig. S1 [30]. All funnel plots were qualitatively symmetrical suggesting no publication bias. Similarly, Egger's tests on testosterone, LH, FSH, CRP, and insulin had p > 0.05, thus we were less likely to miss smaller studies (Fig. S1) [30].

## Discussion

**Fig. 2:**
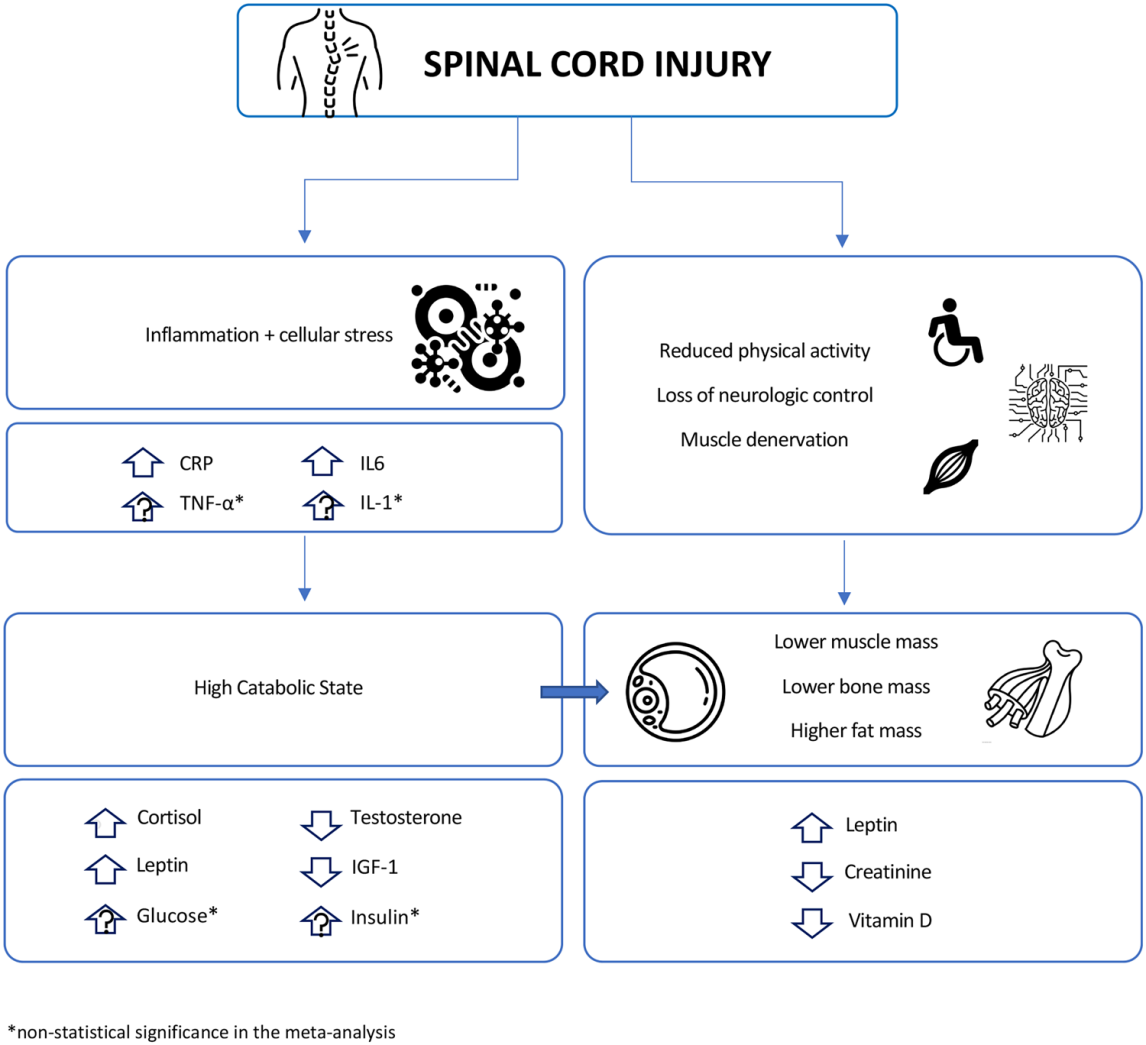
Schematic diagram of the physiologic changes in spinal cord injury

Determining the differences in inflammatory markers and endocrinological profiles of individuals with SCI from ABI could lead to a better understanding of the physiologic changes after SCI and could aid in anticipating complications, and prevention of secondary health outcomes, and rehospitalizations for this high-risk group. We showed that individuals with SCI have higher inflammatory marker levels (CRP and IL-6) compared to ABI. Moreover, we also observed lower creatinine and vitamin D in SCI compared to ABI, which points to changes in body composition. For hormones, total testosterone and IGF-1 were lower in individuals with SCI compared to ABI, while cortisol and leptin were found to be higher. Thus, individuals with SCI showed evidences of chronic inflammation, accelerated muscle and bone loss, and endocrinological alterations of anabolic and catabolic hormones when compared to ABI (Table 3 and Fig. 2).

**Table 3:** Summary of findings of the meta-analysis

**Outcome**	**SCI**	**ABI**
**Glucose and Insulin Profile**		
Insulin	no significant difference	
Glucose		
**Inflammation Profile**		
C-reactive protein	elevated in SCI*	**−**
High sensitivity C-reactive protein	no significant difference	
Interleukin 6	elevated in SCI*	**−**
Tumor necrosis factor-alpha		
**Creatinine Profile**		
Creatinine	decreased in SCI*	**−**
**Vitamin D Profile**		
25(OH)D	decreased in SCI*	**−**
1,25(OH)D	no significant difference	
**Hormone and Growth Factors Profile**		
Total Testosterone	decreased in SCI*	**−**
Free Testosterone		
Thyroid stimulating hormone	no significant difference	
Triiodothyroxine	no significant difference	
Luteinizing hormone	no significant difference	
Follicle stimulating hormone	no significant difference	
Growth hormone	no significant difference	
Cortisol	elevated in SCI*	**−**
Adrenocorticotropic hormone	no significant difference	
Adiponectin	no significant difference	
Aldosterone	no significant difference	
Insulin-like Growth Factor 1	decreased in SCI*	**−**
Leptin	elevated in SCI*	**−**
Prolactin	no significant difference	
Parathyroid hormone	no significant difference	
Sex hormone binding globulin		

^*^ significant WMD values when compared to the ABI

Cytokines are proteins that originate from immune cells, specifically macrophages, and monocytes, at the place of inflammation [37]. We found higher levels of pro-inflammatory cytokines in SCI compared to ABI, particularly CRP and IL-6, which is consistent with the findings from other studies [38, 39]. CRP is known to be an acute phase reactant found at higher levels during the early stages of injury, infection, or other inflammatory stimuli [11]. IL-6 is another cytokine involved in the innate and adaptive immune responses which mediates CRP production [11]. Thus, we find that the injury leads to prolonged higher baseline inflammation levels. There may be at least three plausible explanations for this findings. First, inflammation is the expected physiological response to recurrent urinary, skin, and respiratory infections that are common among individuals with SCI [10]. Second, individuals with SCI are predisposed to higher fat accumulation. Obesity and overweight were observed at rates of 29.9% and 65.8% in SCI and were higher compared to ABI resulting in an increased accumulated volume of adipose tissue [40, 41]. Higher adiposity leads to the release of proinflammatory mediators such as adipokines IL-6 and TNF-α. [42]. Third, there is a close relationship between inflammation and the endocrinologic profile in the SCI group, particularly regarding glucocorticoids and corticotropins [13, 20]. Proinflammatory cytokines can upregulate the hypothalamus–pituitary–adrenal (HPA) axis [13, 20]. A chronic imbalance within the HPA axis in conjunction with hormonal dysregulation may result in immune dysfunction. In an overactivated state of the HPA axis, the corticotropin hormone from the hypothalamus could lead to the release of ACTH from the pituitary gland, followed by the release of glucocorticoids such as cortisol [20]. Overproduction of glucocorticoids may provoke immunosuppression [20]. Such mechanisms could explain the elevated levels of cortisol in individuals with SCI found in our results and other studies [43–45]. Most of the studies, show increased cortisol levels in acute stage of the injury, however, our findings showed also chronically elevated cortisol levels, possibly due to the chronic low-grade inflammatory state in SCI individuals [20]. Furthermore, the adrenal gland which is under the control of the sympathetic trunk from the thoracolumbar spine could be damaged after SCI injury and may affect the balance of steroids hormones [20].

Differences between SCI and ABI individuals regarding sex steroids were also identified in our study. We found lower testosterone levels in individuals with SCI. The literature suggests that the sympathetic innervation of lymphoid organs could also be affected after injury [20]. Low levels of testosterone were observed alongside a high prolactin level [46] and low LH [47], which may suggest a dysfunction of the HPA. In our analysis, two studies observed lower LH in individuals with SCI, but pooled estimates of 15 studies did not reveal any statistically significant difference. Aside from the injury, low levels of testosterone were also associated with several coexisting factors, including age, medication use, obesity, and other clinical comorbidities [21, 48]. Furthermore, hypogonadism has also been reported to be more prevalent amongst SCI individuals compared to age-matched ABI [49].

Moreover, individuals with SCI have altered body composition, specifically lower muscle mass and higher adiposity, when compared to the general population. These conditions are compatible with sarcopenic obesity. Sarcopenic obesity is commonly seen in the elderly and in cancer patients [50, 51] and has been described in the SCI population [52]. In SCI, etiology for sarcopenic obesity is multifactorial and it is characterized by increased leptin levels, low vitamin D, and lower muscle mass, using creatine as a surrogate measure [53]. Creatinine as a metabolite of muscle breakdown is respectively decreased in chronic SCI individuals who have reduced muscle mass due to the increased muscle loss and atrophy from the denervation and reduced physical activity after the injury [54, 55]. Though, SCI individuals experience not only muscle loss but also bone loss. Vitamin D is not only essential for calcium absorption and metabolism but also for bone mineralization which is directly associated with bone mineral density (BMD) [56–61]. SCI individuals show lower vitamin D levels compared to the general population. Moreover, the lack of weight-bearing, diminished limb use, and chronic inflammation associated with the injury also contributes to higher bone loss and bone turnover. Several studies have established these osteopenic changes and this is considered as one of the major health conditions needing attention in this group [62, 63]. Additionally, Vitamin D deficiency has also been linked to other pathological conditions, including autoimmune and inflammatory diseases [64]. Vitamin D, is responsible for decreasing the production of type 1 proinflammatory cytokines and enhancing the generation and activation of type 2 anti-inflammatory cytokines, as well as, T-regulatory cells and tolerogenic dendritic cells [64].

Furthermore, we found lower IGF-1 levels in SCI population compared to the general population, similar to other studies [65, 66]. IGF-1 promotes normal bone and muscle growth [18]. Decreased levels of IGF-1 have been related to the presence of skeletal muscle atrophy and higher fat mass accumulation [8]. A decline in IGF-1 may be also indicative of the development of sarcopenia, which is characterized by a progressive and general loss of skeletal muscle mass and strength [18]. Moreover, we also found higher leptin levels in SCI individuals, similar to other studies [8, 67, 68]. Leptin is released by adipose tissue and past studies have confirmed higher fat accumulation in SCI individuals explaining the higher leptin levels in this group [8]. Leptin levels are 32% higher in persons with SCI compared to ABI [8].

Due to body composition changes and an associated poorer lifestyle after the injury individuals with SCI have also a younger onset and higher incidence of diabetes compared to the general population [69–71]. Furthermore, testosterone deficiency has been associated with higher diabetes predisposition in men [72, 73]. Nonetheless, our results did not show any significant differences in glucose and insulin between these two populations. This can be potentially explained based on three grounds. First, the majority of the studies reported only fasting glucose and insulin levels. Second, in most of the studies in which these outcomes where measured insulin resistant or glucose intolerant individuals were excluded. Lastly, further analysis could not be done as diet, exercise, and antidiabetic medication were not reported—a possible explanation for the high heterogeneity of our pooled estimates. A potential solution would be the use of fasting glucose in combination with 2-h post load glucose (2 h-PG) and hemoglobin A1c (HbA1c) as diagnostic measures, which has proven to be more efficient in predicting the risk of incidence of diabetes and should be considered in future studies [74].

Overall, there are significant differences in inflammatory and endocrine profiles in individuals with SCI compared to the general population. These differences can be directly associated with SCI or derived from secondary health conditions developed post-injury. Alternatively, they could also be viewed as the product of a normal compensatory process as these individuals transition into a new equilibrium. In the initial phases of SCI, compensation could be characterized by intrinsic functional compensation. However, over time, structural changes may occur in order to achieve metabolic homeostasis [75]. As an example, we can expect lower creatinine levels in the SCI population because of reduced muscle mass as a consequence of decreased levels of physical activity and muscle denervation. Nonetheless, decreased levels of anabolic hormones after SCI could also be responsible for the deterioration in body composition or other related metabolic profile disorders and lead to a decreased capacity of cellular repair and maintenance of lean muscle mass and strength [8]. Moreover, since SCI individuals tend to age prematurely, we anticipate a decline of several anabolic and somatotropic hormones, especially sex hormones [17, 18].

Our review synthesized inflammatory and endocrine markers to characterize the physiologic profile in SCI. To the extent of our knowledge, this systematic review and meta-analysis is the first in the literature that focused on inflammatory markers, hormones, and other related metabolites in SCI. Most of the reviews have been focused on specific biomarkers, no systematic literature search, or have had no quantitative synthesis. We used state-of-the-art search syntax and used meta-analysis tools to compute for weighted mean difference and investigate heterogeneity. We collected information on biomarkers from studies which in their majority contained age-and-sex matched individuals. Furthermore, most research in SCI has been based on small sample size resulting in inconsistent effect estimates across studies. We pooled results from different studies to increase the statistical power and precision of the effect estimates. Finally, we provided an absolute value on the difference that could be useful in future research.

However, our analysis had several limitations that need to be considered while interpreting the results. First, our analysis was based on cross-sectional studies. These studies are snapshots of a particular point in time and the directionality of the exposure (injury) and outcome (endocrine and inflammation markers) are difficult to establish. Second, we pooled results from different studies with varying primary outcomes. This may have contributed to high heterogeneity in our findings. We performed meta-regression, subgroup analysis, and leave-one-out, albeit still insufficient in explaining the high heterogeneity. Third, the pooled estimates were not adjusted for lifestyle factors which include physical activity, smoking, medication use, and nutritional intake. Studies did not provide standardized reporting of such lifestyle factors precluding further exploration. Fourth, results were also mostly based on SCI individuals with chronic injury. We cannot speculate at what time point after the injury the physiological changes occur. Finally, our findings are based mostly on males and North American cohorts, which may not be generalizable to other populations.

Future studies should focus on other biomarkers that elucidate the understanding of the physiological changes that SCI carries and optimize prognosis and diagnosis of specific health conditions that derive from the primary injury. For instance, given the fact that the SCI population has a higher risk of developing CVD's, it is important to mention that even though hsCRP, which is indicated specifically for cardiovascular risk assessment, because of its superior assay precision and accuracy, it is nonetheless much less studied than CRP, or less encountered due to bias by indication [42]. Another example is the use of cystatin-c simultaneously with creatinine to estimate renal function in SCI individual, since cystatin-c is not a marker correlated to muscle mass [76]. Furthermore, biomarkers are dynamic and capture a health status at a specific point in time. Therefore, more longitudinal studies are needed to reflect changes in biomarkers over time. In addition, given the individual objectives and research focus of most studies, these were only conducted in males, leading to a lack of female-specific and female-specific risk factors studies. Sex plays a major role in the differences between inflammatory markers and hormones in ABI. For example, few studies have ventured into estradiol and estrogen-derivative hormone levels in SCI and its neuroprotective effects. Estrogen has been shown to increase revascularization, reduce inflammation, reduce oxidative damage, and downregulate the apoptotic pathways in some neurologic conditions [77–79]. A few studies have also shown neuroprotective effects of estrogen supplementation in SCI animal models, although the translation of it into clinical use is yet to be explored. Because few women are involved in most of the studies, clinical trials on the use of estrogen is made more challenging. The underrepresentation of females in SCI conducted studies further emphasizes the need to adopt more sex- and gender-sensitive research frameworks to explore determinant of health in females [80]. Finally, post-injury rehabilitation aims to train gross and fine motor function, and further increase independence in activities of the daily living [81]. Future studies should explore the association between rehabilitation aspects (functional recovery, mobility) and endocrinological and inflammatory profiles.

## Conclusions

SCI results in dramatic physiological changes as a direct result of the injury or by consequence of secondary condition impairments caused by the injury. Our systematic review has shown that individuals with SCI have a higher level of inflammation and significant endocrinological changes reflected not only in higher muscle and bone loss but also in the presence of other metabolic diseases. These findings are aspects of the physiological profile of the SCI population, which need to be considered in anticipating the medical problems and optimizing medical care in this group. As metabolic and endocrine function is altered in SCI individuals, regular screening on diabetes, osteoporosis and hypogonadism is recommended to always be included in patient management guidelines [82]. Moreover, we know that SCI individuals undergo a compensatory process in order to reach metabolic homeostasis, these changes however, do not necessarily indicate the presence of a pathology and could be considered as ''new normal''. Thus, the present results could also elucidate a pathway into adjusting biomarkers reference ranges personalized for individuals with SCI. A fact that is currently being explored by some research groups by creating an adjusted (i.e., age, gender, disability) normal reference ranges for laboratory values (such as the ongoing Swiss BioRef project). Lastly, it is clear that blood biomarkers assessment is of major clinical importance for diagnosis and prognosis, especially within populations at risk such as SCI, therefore, it is important to consider in a parallel approach the use of epigenetic biomarkers. In the CNS, emerging evidence has shown that epigenetic regulation plays a critical role in numerous pathological and physiological processes, such as proliferation, differentiation and regeneration. SCI comprises multiple epigenetic landmark alterations, identifying these could lead to the development of clinical solutions that promote the mechanisms of SCI recovery [83]. However, currently validation of epigenetic biomarkers specific for SCI is not available. Further research should focus on their application in SCI and its related secondary health conditions.

## Supplementary Information

Below is the link to the electronic supplementary material.

Supplementary file1 (DOCX 1616 KB)

## Abbreviations

ABI: Able-bodied individuals
ACTH: Adrenocorticotropic hormone
BMD: Bone mineral density
CI: Confidence interval
CRP: C-reactive protein
CVD: Cardio vascular disease
FSH: Follicle-stimulating hormone
GH: Growth hormone
HbA1c: Hemoglobin A1c (glycosylated hemoglobin)
HPA: Hypothalamus-pituitary-adrenal
hsCRP: High sensitivity c-reactive protein
IGF-1: Insulin-like growth factor 1
IL-6: Interleukin-6
LH: Luteinizing hormone
SHBG: Sex hormone-binding globulin
PTH: Parathyroid hormone
SCI: Spinal cord injury
SI: International system of units
TNF-alpha: Tumor necrosis factor alpha
TSH: Thyroid-stimulating hormone
T3: Triiodothyronine
WMD: Weighted mean difference
2 h-PG: 2 hour postload glucose
