# Supplementary material for: Endocrinological and inflammatory markers in individuals with spinal cord injury: A systematic review and meta-analysis
Source: Rev Endocr Metab Disord. 2022 Aug 18;23(5):1035–50. doi: 10.1007/s11154-022-09742-9 (PMC9515048; doi:10.1007/s11154-022-09742-9)
Supplement: Supplementary file 1 — Supplementary file1 (DOCX 1616 KB) [file 11154_2022_9742_MOESM1_ESM.docx]

**Title**

Endocrinological and inflammatory markers in individuals with spinal cord injury: A systematic review and meta-analysis

**Journal**

Reviews in Endocrine and Metabolic Disorders

**Authors**

Gabriela Boehl^1^*, Peter Francis Raguindin^1,2,3^*, Ezra Valido^1,8^, Alessandro Bertolo^1,7^, Oche Adam Itodo^1,2,3^, Beatrice Minder^4^, Patricia Lampart^5^, Anke Scheel-Sailer^5^, Alexander Leichtle^6^, Marija Glisic^1,2^, Jivko Stoyanov^1,2^

**Author Affiliations**

^1^ Swiss Paraplegic Research, Nottwil, Switzerland

^2^Institute of Social and Preventive Medicine, University of Bern, Switzerland

^3^Graduate School for Health Sciences, University of Bern

^4^Public Health & Primary Care Library, University Library of Bern, University of Bern, Switzerland

^5^Swiss Paraplegic Centre, Nottwil, Switzerland

^6^University Institute of Clinical Chemistry, Inselspital, Bern University Hospital and University of Bern, Switzerland

^7^Department of Orthopedic Surgery, University of Bern, Bern Inselspital, Bern, Switzerland

^8^Department of Health Sciences and Medicine, University of Lucerne, Switzerland

*Denotes equal contribution

**Corresponding author**:

Jivko Stoyanov, PhD

Swiss Paraplegic Research

Guido A. Zäch Str. 4,

6207 Nottwil, Switzerland

[jivko.stoyanov@paraplegie.ch](mailto:jivko.stoyanov@paraplegie.ch)

ORCID 0000-0001-6842-6284

**Search strategy**

**Embase**

*(P1 and P2) AND (O1 or O2 or O3 or O4 or O5 or O6 or O7 or O8 or O9 or O10 or O11) AND Limits*

| *P1* SCI |
| --- |
| ('spinal cord injury'/exp OR 'cervical spine injury'/de OR 'spinal cord ischemia'/de OR 'paraplegia'/de OR 'spastic paraplegia'/de OR 'quadriplegia'/de OR 'spinal dysraphism'/de OR ('injury'/exp AND 'spinal cord'/exp) OR (((spine or spinal) NEAR/3 (injur* or trauma* or damag*)) OR ('spinal cord' NEAR/3 (disease* or contusion* or laceration* or transection* or lesion* or trauma* or ischemi* or ischaemi*)) OR (myelopath* NEAR/3 (trauma* or post-trauma* or posttrauma*)) OR ((spine or spinal or vertebra*) NEAR/3 (fracture* or trauma* or injur* or damage* or wound*)) OR SCI-group* OR 'central cord injury syndrome*' OR 'central cord syndrome*' OR 'central spinal cord syndrome*' OR 'cauda equine syndrome*' OR 'anterior cord syndrome*' OR 'conus medullaris syndrome*' OR 'Brown Sequard' OR paraplegi* OR quadriplegi* OR tetraplegi*):ab,ti,kw) |
| *P2* Able-bodied control group |
| (('general population*' OR (able* NEAR/1 bod*) OR (AB NEAR/1 (group* OR individual* OR participant*)) OR ((healthy OR referen* OR subject* OR age-matched OR gender-matched) NEAR/5 (control OR controls OR group OR adult*)) OR ((reference*) NEAR/3 (sampl* OR value*))):ab,ti,kw) |
| *O1* Lipid Biomarkers |
| ('lipid blood level'/exp OR 'low density lipoprotein cholesterol'/de OR 'high density lipoprotein cholesterol'/exp OR 'lipoprotein'/de OR 'triacylglycerol'/de OR 'dyslipidemia'/de OR (((lipid* OR cholester* OR triacylglycerol* OR triglyceride* OR 'fatty acid*' OR HDL* OR LDL* OR VLDL* OR VHDL*) NEAR/6 (level* OR blood OR serum OR plasma* OR concentration* OR total)) OR hypercholesterol* OR dyslipidemi* OR dyslipidaemi* OR lipoprotein*):ab,ti) |
| *O2* Glucose / Insulin |
| ('insulin response'/exp OR 'glucose blood level'/exp OR 'glucose metabolism'/exp OR 'insulin blood level'/de OR 'insulin sensitivity'/de OR hyperinsulinism/exp OR 'impaired glucose tolerance'/de OR 'impaired fasting glucose'/de OR 'hyperglycemia'/de OR 'fasting glucose'/de OR 'glycosylated hemoglobin'/exp OR 'glycemic control'/de OR 'homa ir'/exp OR (((glucose OR sugar OR insulin*) NEAR/3 (level* OR blood OR serum OR plasma* OR concentration* OR tolerance OR intolerance OR sensitiv* OR insensitiv* OR resistan* OR response OR dependen* OR homeosta*)) OR hypoglycemi* OR hypoglycaemi* OR hyperglycemi* OR hyperglycaemi* OR antihyperglycemi* OR fasting NEAR/2 glucose OR 'glycated hemoglobin*' OR 'glycated haemoglobin*' OR 'glycosylated haemoglobin*' OR 'glycosylated hemoglobin*' OR HbA1c OR 'Hb A1c' OR 'hemoglobin A1c' OR 'haemoglobin A1c' OR HOMA-IR OR glycaem* OR glycem* OR glucosaemia OR glucosemia OR hyperinsulin* OR hypoinsulin* OR insulinem* OR insulinaem*):ab,ti) |
| *O3* Inflammation marker |
| ((inflammation/de AND (marker/de OR 'C reactive protein'/exp OR 'high sensitivity c reactive protein'/de OR cytokine/de OR fibrinolysis/exp OR 'tumor necrosis factor alpha'/exp)) OR 'c reactive protein blood level'/de OR 'nicotinamide phosphoribosyltransferase'/de OR 'chronic inflammation'/exp OR 'oxidative stress'/de OR ((inflammat* NEAR/3 (chronic* OR marker* OR biomarker* OR interleukin* OR CRP OR Hs-CRP OR 'c reactive' OR cytokine* OR fibrinolys* OR fibrinogenlys* OR 'tumor necrosis factor' OR tnf)) OR (oxidative NEAR/3 stress*)):ab,ti) |
| *O4* Kidney function |
| ('kidney function'/exp OR 'neurogenic bladder'/de OR 'creatinine blood level'/de OR 'creatinine clearance'/de OR 'cystatin C'/de OR 'urea nitrogen blood level'/de OR 'glomerulus filtration rate'/exp OR ((kidney OR renal) NEAR/2 (function*)) OR 'neurogenic bladder' OR (urinary NEAR/3 (infect* OR condition*)) OR creatinine* OR 'cystatin C' OR CysC OR Cys-C OR ((blood OR plasma OR serum) NEAR/3 (nitrogen)) OR (glomerul* NEAR/1 filtration NEAR/1 rate*):ab,ti) |
| *O5* Endocrine system |
| ('endocrine system'/exp OR 'vasopressin'/de OR 'hormone blood level'/exp OR 'follitropin'/de OR 'luteinizing hormone'/de OR 'growth hormone'/de OR 'parathyroid hormone'/de OR 'thyroid disease'/de OR 'hyperthyroidism'/exp OR 'hypothyroidism'/exp OR 'thyroid hormone'/de OR 'thyroxine'/de OR 'thyrotropin'/de OR 'steroid hormone'/exp OR 'gonadotropin'/de OR 'estradiol'/de OR (vasopressin* OR ((anti-diuretic OR antidiuretic OR follicle-stimulating OR luteinizing OR luteinising OR growth OR parathyroid OR thyroid-stimulati*) AND (hormone OR hormones)) OR parathormon* OR follitropin* OR lutotropin* OR somatotrop* OR parathyrin* OR thyrotrophin* OR thyrotropin OR hypothyroid* OR hyperthyroid* OR thyroid dysfunction* OR triiodothyronin* OR thyroxin* OR TSH OR T4 OR T3 OR thyroid failure* OR thyroid disease* OR steroid* OR corticosteroid* OR corticoid* OR cortisone OR cortisol OR estrogen* OR oestrogen* OR androgen* OR testosterone* OR gonadotropin* OR estradiol* OR oestradiol*):ab,ti) |
| *O6* Electrolytes |
| ('electrolyte'/exp OR 'electrolyte disturbance'/exp OR 'electrolyte urine level'/exp OR 'electrolyte blood level'/exp OR 'mineral blood level'/exp OR 'calcium'/de OR 'calcium urine level'/exp OR 'phosphorus'/de OR 'phosphate'/de OR 'magnesium'/de OR 'osteoporosis'/exp OR 'osteopenia'/de OR 'bone density'/exp OR 'bone mineralization'/de OR (electrolyt* OR sodium OR potassium OR kalium OR calcium OR phosphor* OR magnesium OR natrium OR osteoporo* OR osteopenia* OR ((bone OR osseous) NEAR/3 (densit* OR mineral* OR loss)) OR hyponatraemi* OR hyponatriaemi* OR hyponatremi* OR hyponatriemi* OR hypernatraemi* OR hypernatriaemi* OR hypernatriemi* OR hypernatriemi* OR hypokalaemi* OR hypokaliaemi* OR hypokaliemi* OR hypokalemi* OR hypercalcemi* OR hypercalcaemi* OR hypocalcemi* OR hypocalcaemi* OR hyperphosphatemia* OR hyperphosphataemi* OR hypophosphataemi* OR hypophosphatemia* OR hypomagnesemia* OR hypomagnesaemia*):ab,ti) |
| *O7* Red/white blood cells, hemoglobin |
| ('blood cell'/exp OR 'iron blood level'/de OR 'hematocrit'/de OR 'transferrin'/de OR 'ferritin'/de OR 'iron binding protein'/exp OR 'erythropoietin'/de OR 'soluble transferrin receptor'/de OR 'hepcidin'/de OR 'haptoglobin'/de OR 'unsaturated iron binding capacity'/de OR 'erythrocyte'/exp OR 'erythrocyte protoporphyrin'/de OR 'erythrocyte parameters'/exp OR 'erythrocyte count'/de OR 'red cell distribution width'/de OR 'erythrocyte volume'/de OR 'hemoglobin'/exp OR 'hemoglobin blood level'/de OR (blood OR serum OR 'red cell*' OR erythrocyte* OR leucocyte* OR lymphocyte* OR monocyte* OR neutrophil* OR basophil* OR eosinophil* OR haematocrit OR hematocrit OR transferrin OR transferrins OR apoferritin* OR ferritin* OR erythropoieti* OR hematopoie* OR hemopoie* OR haematopoie* OR haemopoie* OR hepcidin* OR haptoglobin* OR hemoglobin* OR haemoglobin* OR hemoglobulin* OR haemoglobulin* OR ferrohaemoglobin* OR ferrohemoglobin* OR iron):ab,ti) |
| *O8* Coagulation |
| ('blood clotting'/exp OR 'blood clotting disorder'/exp OR 'blood clotting factor'/exp OR 'blood clotting parameters'/exp OR 'vitamin K group'/exp OR (fibrinogen* OR fibrinoly* OR ((blood) NEAR/3 (clotting OR clot OR clots OR coagulat*)) OR 'clotting time*' OR 'coagulation factor*' OR 'coagulation time*' OR 'coagulation cascade*' OR 'thrombin time*' OR platelets OR INR OR prothrombi* OR thrombinogen* OR thrombogen* OR 'partial thromboplastin time*' OR thrombinogen* OR hemostat* OR haemosta* OR 'vitamin K'):ab,ti) |
| *O9* Vitamin D |
| ('vitamin D'/exp OR 'vitamin D deficiency'/de OR 'calcidiol 1 monooxygenase'/de OR ('vitamin D' OR '25-hydroxyvitamin D' OR 25-OH OR 25-hydroxy* OR calcidiol OR calcitriol):ab,ti) |
| *O10* Liver function |
| ('liver function'/exp OR 'liver function test'/de OR 'aspartate aminotransferase'/de OR 'alanine aminotransferase'/de OR 'alkaline phosphatase'/de OR 'gamma glutamyltransferase'/de OR 'albumin'/de OR 'serum albumin'/de OR (((liver OR hepatic OR hepatocyte OR hepatocellular) NEAR/3 (function* OR inflammat*)) OR 'aspartate aminotransferase' OR 'aspartate transaminase' OR 'alanine aminotransferase' OR 'alanine transaminase' OR 'alkaline phosphatase' OR 'gamma glutamyltransferase' OR 'gamma glutamyl transferase' OR 'gamma glutamyl transpeptidase' OR 'gamma glutamyltranspeptidase' OR albumin*):ab,ti) |
| *O11* Biomarkers |
| ('marker'/exp OR 'biological marker'/de OR 'biochemical marker'/de OR (marker OR markers OR biomarker*):ab,ti) |
| Limits |
| Exclusion: NOT ([animals]/lim NOT [humans]/lim) NOT ([Conference Abstract]/lim OR [Letter]/lim OR [Note]/lim OR [Editorial]/lim) |

**Medline (Ovid)**

| *P1* SCI |
| --- |
| (exp Spinal Cord Injuries/ OR exp Spinal Cord Ischemia/ OR exp Paraplegia/ OR Quadriplegia/ OR Spinal Dysraphism/ OR (((spine OR spinal) ADJ3 (injur* OR trauma* OR damag*)) OR (spinal cord ADJ3 (disease* OR contusion* OR laceration* OR transection* OR lesion* OR trauma* OR ischemi* OR ischaemi*)) OR (myelopath* ADJ3 (trauma* OR post-trauma* OR posttrauma*)) OR ((spine OR spinal OR vertebra*) ADJ3 (fracture* OR trauma* OR injur* OR damage* OR wound*)) OR SCI-group* OR central cord injury syndrome* OR central cord syndrome* OR central spinal cord syndrome* OR cauda equine syndrome* OR anterior cord syndrome* OR conus medullaris syndrome* OR Brown Sequard OR paraplegi* OR quadriplegi* OR tetraplegi*).ab,ti,kf.) |
| *P2* Able-bodied control group |
| ((general population* OR (able* ADJ1 bod*) OR (AB ADJ1 (group* OR individual* OR participant*)) OR ((healthy OR referen* OR subject* OR age-matched OR gender-matched) ADJ5 (control OR controls OR group OR adult*)) OR ((reference*) ADJ3 (sampl* OR value*))).ab,ti,kw.) |
| *O1* Lipid Biomarkers |
| (exp Lipids/bl OR Cholesterol, LDL/ OR Cholesterol, HDL/ OR exp Lipoproteins/ OR exp Triglycerides/ OR exp Dyslipidemias/ OR (((lipid* OR cholester* OR triacylglycerol* OR triglyceride* OR fatty acid* OR HDL* OR LDL* OR VLDL* OR VHDL*) ADJ6 (level* OR blood OR serum OR plasma* OR concentration* OR total)) OR hypercholesterol* OR dyslipidemi* OR dyslipidaemi* OR lipoprotein*).ab,ti.) |
| *O2* Glucose / Insulin |
| (exp Insulin Resistance/ OR Blood Glucose/ OR Glucose/ OR Insulin/bl OR Hyperinsulinism/ OR Glucose Intolerance/ OR Hyperglycemia/ OR Glycated Hemoglobin A/ OR (((glucose OR sugar OR insulin*) ADJ3 (level* OR blood OR serum OR plasma* OR concentration* OR tolerance OR intolerance OR sensitiv* OR insensitiv* OR resistan* OR response OR dependen* OR homeosta*)) OR hypoglycemi* OR hypoglycaemi* OR hyperglycemi* OR hyperglycaemi* OR antihyperglycemi* OR fasting ADJ2 glucose OR glycated hemoglobin* OR glycated haemoglobin* OR glycosylated haemoglobin* OR glycosylated hemoglobin* OR HbA1c OR Hb A1c OR hemoglobin A1c OR haemoglobin A1c OR HOMA-IR OR glycaem* OR glycem* OR glucosaemia* OR glucosemia* OR hyperinsulin* OR hypoinsulin* OR insulinem* OR insulinaem*).ab,ti.) |
| *O3* Inflammation marker |
| ((Inflammation/ AND (exp Biomarkers/ OR C Reactive Protein/ OR exp Cytokines/ OR Fibrinolysis/ OR Tumor Necrosis Factor-alpha/)) OR C-Reactive Protein/bl OR Nicotinamide Phosphoribosyltransferase/ OR Oxidative Stress/ OR ((inflammat* ADJ3 (chronic* OR marker* OR biomarker* OR interleukin* OR CRP OR Hs-CRP OR c reactive OR cytokine* OR fibrinolys* OR fibrinogenlys* OR tumor necrosis factor OR tnf)) OR (oxidative ADJ3 stress*)).ab,ti.) |
| *O4* Kidney function |
| (Kidney/ OR exp Kidney Function Tests/ OR Urinary Bladder, Neurogenic/ OR Creatinine/ OR Cystatin C/ OR Blood Urea Nitrogen/ OR Glomerular Filtration Rate/ OR ((kidney OR renal) ADJ2 (function*)) OR neurogenic bladder OR (urinary ADJ3 (infect* OR condition*)) OR creatinine* OR cystatin C OR CysC OR Cys-C OR ((blood OR plasma OR serum) ADJ3 (nitrogen)) OR (glomerul* ADJ1 filtration ADJ1 rate*).ab,ti.) |
| *O5* Endocrine system |
| (exp Endocrine System/ OR exp Vasopressins/ OR exp Hormones/bl OR exp Follicle Stimulating Hormone/ OR exp Luteinizing Hormone/ OR exp Growth Hormone/ OR exp Parathyroid Hormone/ OR Thyroid Diseases/ OR exp Hyperthyroidism/ OR exp Hypothyroidism/ OR exp Thyroid Hormones/ OR Thyroxine/ OR exp Thyrotropin/ OR exp Gonadal Steroid Hormones/ OR exp Gonadotropins/ OR exp Estradiol/ OR (vasopressin* OR ((anti-diuretic OR antidiuretic OR follicle-stimulating OR luteinizing OR luteinising OR growth OR parathyroid OR thyroid-stimulati*) AND (hormone OR hormones)) OR parathormon* OR follitropin* OR lutotropin* OR somatotrop* OR parathyrin* OR thyrotrophin* OR thyrotropin OR hypothyroid* OR hyperthyroid* OR thyroid dysfunction* OR triiodothyronin* OR thyroxin* OR TSH OR T4 OR T3 OR thyroid failure* OR thyroid disease* OR steroid* OR corticosteroid* OR corticoid* OR cortisone OR cortisol OR estrogen* OR oestrogen* OR androgen* OR testosterone* OR gonadotropin* OR estradiol* OR oestradiol*).ab,ti.) |
| *O6* Electrolytes |
| (exp Electrolytes/ OR exp Water-Electrolyte Imbalance/ OR exp Calcium Metabolism Disorders/ OR Minerals/bl, ur OR exp Calcium/ OR Phosphorus/ OR exp Phosphates/ OR Magnesium/ OR exp Osteoporosis/ OR exp Bone Diseases, Metabolic/ OR Bone Density/ OR Calcification, Physiologic/ OR (electrolyt* OR sodium OR potassium OR kalium OR calcium OR phosphor* OR magnesium OR natrium OR osteoporo* OR osteopenia* OR ((bone OR osseous) ADJ3 (densit* OR mineral* OR loss)) OR hyponatraemi* OR hyponatriaemi* OR hyponatremi* OR hyponatriemi* OR hypernatraemi* OR hypernatriaemi* OR hypernatriemi* OR hypernatriemi* OR hypokalaemi* OR hypokaliaemi* OR hypokaliemi* OR hypokalemi* OR hypercalcemi* OR hypercalcaemi* OR hypocalcemi* OR hypocalcaemi* OR hyperphosphatemia* OR hyperphosphataemi* OR hypophosphataemi* OR hypophosphatemia* OR hypomagnesemia* OR hypomagnesaemia*).ab,ti.) |
| *O7* Red/white blood cells, hemoglobin |
| (exp Blood Cells/ OR Iron/bl OR Hematocrit/ OR Transferrin/ OR exp Ferritins/ OR exp Iron-Binding Proteins/ OR exp Erythropoietin/ OR Receptors, Transferrin/ OR Hepcidins/ OR Haptoglobins/ OR exp Erythrocytes/ OR exp Blood Cell Count/ OR exp Erythrocyte Count/ OR Erythrocyte Indices/ OR exp Hemoglobins/ OR blood.fs. OR (blood OR serum OR red cell* OR erythrocyte* OR leucocyte* OR lymphocyte* OR monocyte* OR neutrophil* OR basophil* OR eosinophil* OR haematocrit OR hematocrit OR transferrin OR transferrins OR apoferritin* OR ferritin* OR erythropoieti* OR hematopoie* OR hemopoie* OR haematopoie* OR haemopoie* OR hepcidin* OR haptoglobin* OR hemoglobin* OR haemoglobin* OR hemoglobulin* OR haemoglobulin* OR ferrohaemoglobin* OR ferrohemoglobin* OR iron).ab,ti.) |
| *O8* Coagulation |
| (exp Blood Coagulation/ OR exp Blood Coagulation Disorders/ OR exp Blood Coagulation Factors/ OR exp Blood Coagulation Tests/ OR Vitamin K/ OR (fibrinogen* OR fibrinoly* OR ((blood) ADJ3 (clotting OR clot OR clots OR coagulat*)) OR clotting time* OR coagulation factor* OR coagulation time* OR coagulation cascade* OR thrombin time* OR platelets OR INR OR prothrombi* OR thrombinogen* OR thrombogen* OR partial thromboplastin time* OR thrombinogen* OR hemostat* OR haemosta* OR vitamin K).ab,ti.) |
| *O9* Vitamin D |
| (exp Vitamin D/ OR exp Vitamin D Deficiency/ OR 25-Hydroxyvitamin D3 1-alpha-Hydroxylase/ OR (vitamin D OR 25-hydroxyvitamin D OR 25-OH OR 25-hydroxy* OR calcidiol OR calcitriol).ab,ti.) |
| *O10* Liver function |
| (exp Liver/ OR Liver Function Tests/ OR exp Aspartate Aminotransferases/ OR Alanine Transaminase/ OR Alkaline Phosphatase/ OR gamma-Glutamyltransferase/ OR exp Albumins/ OR exp Serum Albumin/ OR (((liver OR hepatic OR hepatocyte OR hepatocellular) ADJ3 (function* OR inflammat*)) OR aspartate aminotransferase OR aspartate transaminase OR alanine aminotransferase OR alanine transaminase OR alkaline phosphatase OR gamma glutamyltransferase OR gamma glutamyl transferase OR gamma glutamyl transpeptidase OR gamma glutamyltranspeptidase OR albumin*).ab,ti.) |
| *O11* Biomarkers |
| (exp Biomarkers/ OR (marker OR markers OR biomarker*).ab,ti,kf) |
| Limits |
| NOT (exp animals/ NOT humans/) NOT (letter OR news OR comment OR editorial OR congres* OR abstract*).pt. |

**PubMed publisher as supplied by publisher**

| *P1* SCI |
| --- |
| (Spinal Cord Injuries[mh] OR Spinal Cord Ischemia[mh] OR Paraplegia[mh] OR Quadriplegia[mh] OR Spinal Dysraphism[mh] OR (((spine[tiab] OR spinal[tiab]) AND (injur*[tiab] OR trauma*[tiab] OR damag*[tiab])) OR (spinal cord[tiab] AND (disease*[tiab] OR contusion*[tiab] OR laceration*[tiab] OR transection*[tiab] OR lesion*[tiab] OR trauma*[tiab] OR ischemi*[tiab] OR ischaemi*[tiab])) OR (myelopath*[tiab] AND (trauma*[tiab] OR post-trauma*[tiab] OR posttrauma*[tiab])) OR ((spine[tiab] OR spinal[tiab] OR vertebra*[tiab]) AND (fracture*[tiab] OR trauma*[tiab] OR injur*[tiab] OR damage*[tiab] OR wound*[tiab])) OR SCI-group*[tiab] OR central cord injury syndrome*[tiab] OR central cord syndrome*[tiab] OR central spinal cord syndrome*[tiab] OR cauda equine syndrome*[tiab] OR anterior cord syndrome*[tiab] OR conus medullaris syndrome*[tiab] OR Brown Sequard[tiab] OR paraplegi*[tiab] OR quadriplegi*[tiab] OR tetraplegi*[tiab])) |
| *P2* Able-bodied control group |
| ((general population*[tiab] OR (able*[tiab] AND (bodied[tiab] OR bodiedness[tiab] OR body[tiab] OR bodies[tiab] OR bodyism[tiab] OR bodiedadult[tiab] OR bodism[tiab])) OR (AB[tiab] AND (group*[tiab] OR individual*[tiab] OR participant*[tiab])) OR ((healthy[tiab] OR referen*[tiab] OR subject*[tiab] OR age-matched[tiab] OR gender-matched[tiab]) AND (control[tiab] OR controls[tiab] OR group[tiab] OR adult*[tiab])) OR ((reference*[tiab]) AND (sampl*[tiab] OR value*[tiab])))) |
| *O1* Lipid Biomarkers |
| ("Lipids/blood"[Mesh] OR Cholesterol, LDL[mh] OR Cholesterol, HDL[mh] OR Lipoproteins[mh] OR Triglycerides[mh] OR Dyslipidemias[mh] OR (((lipid*[tiab] OR cholester*[tiab] OR triacylglycerol*[tiab] OR triglyceride*[tiab] OR fatty acid*[tiab] OR HDL[tiab] OR LDL[tiab] OR VLDL*[tiab] OR VHDL*[tiab]) AND (level*[tiab] OR blood[tiab] OR serum[tiab] OR plasma*[tiab] OR concentration*[tiab] OR total[tiab])) OR hypercholesterol*[tiab] OR dyslipidemi*[tiab] OR dyslipidaemi*[tiab] OR lipoprotein*[tiab])) |
| *O2* Glucose [mh] Insulin |
| (Insulin Resistance[mh] OR Blood Glucose[mh] OR Glucose[mh] OR "Insulin/blood"[Mesh] OR Hyperinsulinism[mh] OR Glucose Intolerance[mh] OR Hyperglycemia[mh] OR Glycated Hemoglobin A[mh] OR (((glucose[tiab] OR sugar[tiab] OR insulin*[tiab]) AND (level*[tiab] OR blood[tiab] OR serum[tiab] OR plasma*[tiab] OR concentration*[tiab] OR tolerance[tiab] OR intolerance[tiab] OR sensitiv*[tiab] OR insensitiv*[tiab] OR resistan*[tiab] OR response[tiab] OR dependen*[tiab] OR homeosta*[tiab])) OR hypoglycemi*[tiab] OR hypoglycaemi*[tiab] OR hyperglycemi*[tiab] OR hyperglycaemi*[tiab] OR antihyperglycemi*[tiab] OR "fasting glucose" OR glycated hemoglobin*[tiab] OR glycated haemoglobin*[tiab] OR glycosylated haemoglobin*[tiab] OR glycosylated hemoglobin*[tiab] OR HbA1c[tiab] OR Hb A1c[tiab] OR hemoglobin A1c[tiab] OR haemoglobin A1c[tiab] OR HOMA-IR[tiab] OR glycaem*[tiab] OR glycem*[tiab] OR glucosaemia*[tiab] OR glucosemia*[tiab] OR hyperinsulin*[tiab] OR hypoinsulin*[tiab] OR insulinem*[tiab] OR insulinaem*[tiab])) |
| *O3* Inflammation marker |
| ((Inflammation[mh] AND (Biomarkers[mh] OR C Reactive Protein[mh] OR Cytokines[mh] OR Fibrinolysis[mh] OR Tumor Necrosis Factor-alpha[mh])) OR "C-Reactive Protein/blood"[Mesh] OR Nicotinamide Phosphoribosyltransferase[mh] OR Oxidative Stress[mh] OR ((inflammat*[tiab] AND (chronic*[tiab] OR marker*[tiab] OR biomarker*[tiab] OR interleukin*[tiab] OR CRP[tiab] OR Hs-CRP[tiab] OR c reactive[tiab] OR cytokine*[tiab] OR fibrinolys*[tiab] OR fibrinogenlys*[tiab] OR tumor necrosis factor[tiab] OR tnf[tiab])) OR (oxidative[tiab] AND stress*[tiab]))) |
| *O4* Kidney function |
| (Kidney[mh] OR "Kidney Function Tests"[Mesh] OR Urinary Bladder, Neurogenic[mh] OR Creatinine[mh] OR Cystatin C[mh] OR Blood Urea Nitrogen[mh] OR Glomerular Filtration Rate[mh] OR (("kidney function*"[tiab] OR "renal function*"[tiab])) OR neurogenic bladder[tiab] OR (urinary[tiab] AND (infect*[tiab] OR condition*[tiab])) OR creatinine*[tiab] OR cystatin C[tiab] OR CysC[tiab] OR Cys-C[tiab] OR ((blood[tiab] OR plasma[tiab] OR serum[tiab]) AND (nitrogen[tiab])) OR (glomerul*[tiab] AND filtration[tiab] AND rate*[tiab])) |
| *O5* Endocrine system |
| (Endocrine System[mh] OR Vasopressins[mh] OR "Hormones/blood"[Mesh] OR Follicle Stimulating Hormone[mh] OR Luteinizing Hormone[mh] OR Growth Hormone[mh] OR Parathyroid Hormone[mh] OR Thyroid Diseases[mh] OR Hyperthyroidism[mh] OR Hypothyroidism[mh] OR Thyroid Hormones[mh] OR Thyroxine[mh] OR Thyrotropin[mh] OR Gonadal Steroid Hormones[mh] OR Gonadotropins[mh] OR Estradiol[mh] OR (vasopressin*[tiab] OR ((anti-diuretic[tiab] OR antidiuretic[tiab] OR follicle-stimulating[tiab] OR luteinizing[tiab] OR luteinising[tiab] OR growth[tiab] OR parathyroid[tiab] OR thyroid-stimulati*[tiab]) AND (hormone[tiab] OR hormones[tiab])) OR parathormon*[tiab] OR follitropin*[tiab] OR lutotropin*[tiab] OR somatotrop*[tiab] OR parathyrin*[tiab] OR thyrotrophin*[tiab] OR thyrotropin[tiab] OR hypothyroid*[tiab] OR hyperthyroid*[tiab] OR thyroid dysfunction*[tiab] OR triiodothyronin*[tiab] OR thyroxin*[tiab] OR TSH[tiab] OR T4[tiab] OR T3[tiab] OR thyroid failure*[tiab] OR thyroid disease*[tiab] OR steroid*[tiab] OR corticosteroid*[tiab] OR corticoid*[tiab] OR cortisone[tiab] OR cortisol[tiab] OR estrogen*[tiab] OR oestrogen*[tiab] OR androgen*[tiab] OR testosterone*[tiab] OR gonadotropin*[tiab] OR estradiol*[tiab] OR oestradiol*[tiab])) |
| *O6* Electrolytes |
| (Electrolytes[mh] OR Water-Electrolyte Imbalance[mh] OR Calcium Metabolism Disorders[mh] OR "Minerals/blood"[Mesh] OR "Minerals/urine"[Mesh] OR Calcium[mh] OR Phosphorus[mh] OR Phosphates[mh] OR Magnesium[mh] OR Osteoporosis[mh] OR Bone Diseases, Metabolic[mh] OR Bone Density[mh] OR Calcification, Physiologic[mh] OR (electrolyt*[tiab] OR sodium[tiab] OR potassium[tiab] OR kalium[tiab] OR calcium[tiab] OR phosphor*[tiab] OR magnesium[tiab] OR natrium[tiab] OR osteoporo*[tiab] OR osteopenia*[tiab] OR ((bone[tiab] OR osseous[tiab]) AND (densit*[tiab] OR mineral*[tiab] OR loss[tiab])) OR hyponatraemi*[tiab] OR hyponatriaemi*[tiab] OR hyponatremi*[tiab] OR hyponatriemi*[tiab] OR hypernatraemi*[tiab] OR hypernatriaemi*[tiab] OR hypernatriemi*[tiab] OR hypernatriemi*[tiab] OR hypokalaemi*[tiab] OR hypokaliaemi*[tiab] OR hypokaliemi*[tiab] OR hypokalemi*[tiab] OR hypercalcemi*[tiab] OR hypercalcaemi*[tiab] OR hypocalcemi*[tiab] OR hypocalcaemi*[tiab] OR hyperphosphatemia*[tiab] OR hyperphosphataemi*[tiab] OR hypophosphataemi*[tiab] OR hypophosphatemia*[tiab] OR hypomagnesemia*[tiab] OR hypomagnesaemia*[tiab])) |
| *O7* Red/white blood cells, hemoglobin |
| (Blood Cells[mh] OR "Iron/blood"[Mesh] OR Hematocrit[mh] OR Transferrin[mh] OR Ferritins[mh] OR Iron-Binding Proteins[mh] OR Erythropoietin[mh] OR Receptors, Transferrin[mh] OR Hepcidins[mh] OR Haptoglobins[mh] OR Erythrocytes[mh] OR Blood Cell Count[mh] OR Erythrocyte Count[mh] OR Erythrocyte Indices[mh] OR Hemoglobins[mh] OR "blood" [Subheading] OR (blood[tiab] OR serum[tiab] OR red cell*[tiab] OR erythrocyte*[tiab] OR leucocyte*[tiab] OR lymphocyte*[tiab] OR monocyte*[tiab] OR neutrophil*[tiab] OR basophil*[tiab] OR eosinophil*[tiab] OR haematocrit[tiab] OR hematocrit[tiab] OR transferrin[tiab] OR transferrins[tiab] OR apoferritin*[tiab] OR ferritin*[tiab] OR erythropoieti*[tiab] OR hematopoie*[tiab] OR hemopoie*[tiab] OR haematopoie*[tiab] OR haemopoie*[tiab] OR hepcidin*[tiab] OR haptoglobin*[tiab] OR hemoglobin*[tiab] OR haemoglobin*[tiab] OR hemoglobulin*[tiab] OR haemoglobulin*[tiab] OR ferrohaemoglobin*[tiab] OR ferrohemoglobin*[tiab] OR iron[tiab])) |
| *O8* Coagulation |
| (Blood Coagulation[mh] OR Blood Coagulation Disorders[mh] OR Blood Coagulation Factors[mh] OR Blood Coagulation Tests[mh] OR Vitamin K[mh] OR (fibrinogen*[tiab] OR fibrinoly*[tiab] OR ((blood[tiab]) AND (clotting[tiab] OR clot[tiab] OR clots[tiab] OR coagulat*[tiab])) OR clotting time*[tiab] OR coagulation factor*[tiab] OR coagulation time*[tiab] OR coagulation cascade*[tiab] OR thrombin time*[tiab] OR platelets[tiab] OR INR[tiab] OR prothrombi*[tiab] OR thrombinogen*[tiab] OR thrombogen*[tiab] OR partial thromboplastin time*[tiab] OR thrombinogen*[tiab] OR hemostat*[tiab] OR haemosta*[tiab] OR vitamin K[tiab])) |
| *O9* Vitamin D |
| (Vitamin D[mh] OR Vitamin D Deficiency[mh] OR 25-Hydroxyvitamin D3 1-alpha-Hydroxylase[mh] OR (vitamin D[tiab] OR 25-hydroxyvitamin D[tiab] OR 25-OH[tiab] OR 25-hydroxy*[tiab] OR calcidiol[tiab] OR calcitriol[tiab])) |
| *O10* Liver function |
| (Liver[mh] OR Liver Function Tests[mh] OR Aspartate Aminotransferases[mh] OR Alanine Transaminase[mh] OR Alkaline Phosphatase[mh] OR gamma-Glutamyltransferase[mh] OR Albumins[mh] OR Serum Albumin[mh] OR (((liver[tiab] OR hepatic[tiab] OR hepatocyte[tiab] OR hepatocellular[tiab]) AND (function*[tiab] OR inflammat*[tiab])) OR aspartate aminotransferase[tiab] OR aspartate transaminase[tiab] OR alanine aminotransferase[tiab] OR alanine transaminase[tiab] OR alkaline phosphatase[tiab] OR gamma glutamyltransferase[tiab] OR gamma glutamyl transferase[tiab] OR gamma glutamyl transpeptidase[tiab] OR gamma glutamyltranspeptidase[tiab] OR albumin*[tiab])) |
| *O11* Biomarkers |
| (Biomarkers[mh] OR (marker[tiab] OR markers[tiab] OR biomarker*[tiab])) |
| Limits |
| NOT (animals[mh] NOT humans[mh]) NOT (letter[pt] OR news[pt] OR comment[pt] OR editorial[pt] OR congress[pt] OR abstracts[pt]) AND (publisher[sb] OR inprocess [sb]) |

**Cochrane**

| *P1* SCI |
| --- |
| ((((spine or spinal) NEAR/3 (injur* or trauma* or damag*)) OR ('spinal cord' NEAR/3 (disease* or contusion* or laceration* or transection* or lesion* or trauma* or ischemi* or ischaemi*)) OR (myelopath* NEAR/3 (trauma* or post-trauma* or posttrauma*)) OR ((spine or spinal or vertebra*) NEAR/3 (fracture* or trauma* or injur* or damage* or wound*)) OR SCI-group* OR 'central cord injury syndrome' OR 'central cord syndrome' OR 'central spinal cord syndrome' OR 'cauda equine syndrome' OR 'anterior cord syndrome' OR 'conus medullaris syndrome' OR 'Brown Sequard' OR paraplegi* OR quadriplegi* OR tetraplegi*):ab,ti,kw) |
| *P2* Able-bodied control group |
| ((general NEXT population* OR (able* NEAR/1 bod*) OR (AB NEAR/1 (group* OR individual* OR participant*)) OR ((healthy OR referen* OR subject* OR age-matched OR gender-matched) NEAR/5 (control OR controls OR group OR adult*)) OR ((reference*) NEAR/3 (sampl* OR value*))):ab,ti,kw) |
| *O1* Lipid Biomarkers |
| ((((lipid* OR cholester* OR triacylglycerol* OR triglyceride* OR 'fatty acid*' OR HDL* OR LDL* OR VLDL* OR VHDL*) NEAR/6 (level* OR blood OR serum OR plasma* OR concentration* OR total)) OR hypercholesterol* OR dyslipidemi* OR dyslipidaemi* OR lipoprotein*):ab,ti) |
| *O2* Glucose / Insulin |
| ((((glucose OR sugar OR insulin*) NEAR/3 (level* OR blood OR serum OR plasma* OR concentration* OR tolerance OR intolerance OR sensitiv* OR insensitiv* OR resistan* OR response OR dependen* OR homeosta*)) OR hypoglycemi* OR hypoglycaemi* OR hyperglycemi* OR hyperglycaemi* OR antihyperglycemi* OR fasting NEAR/2 glucose OR 'glycated hemoglobin' OR 'glycated haemoglobin' OR 'glycosylated haemoglobin' OR 'glycosylated hemoglobin' OR HbA1c OR 'Hb A1c' OR 'hemoglobin A1c' OR 'haemoglobin A1c' OR HOMA-IR OR glycaem* OR glycem* OR glucosaemia OR glucosemia OR hyperinsulin* OR hypoinsulin* OR insulinem* OR insulinaem*):ab,ti) |
| *O3* Inflammation marker |
| (((inflammat* NEAR/3 (chronic* OR marker* OR biomarker* OR interleukin* OR CRP OR 'Hs CRP' OR 'c reactive' OR cytokine* OR fibrinolys* OR fibrinogenlys* OR 'tumor necrosis factor' OR tnf)) OR (oxidative NEAR/3 stress*)):ab,ti) |
| *O4* Kidney function |
| (((kidney OR renal) NEAR/2 (function*)) OR 'neurogenic bladder' OR (urinary NEAR/3 (infect* OR condition*)) OR creatinine* OR 'cystatin C' OR CysC OR Cys-C OR ((blood OR plasma OR serum) NEAR/3 (nitrogen)) OR (glomerul* NEAR/1 filtration NEAR/1 rate*):ab,ti) |
| *O5* Endocrine system |
| ((vasopressin* OR ((anti-diuretic OR antidiuretic OR follicle-stimulating OR luteinizing OR luteinising OR growth OR parathyroid OR thyroid-stimulati*) AND (hormone OR hormones)) OR parathormon* OR follitropin* OR lutotropin* OR somatotrop* OR parathyrin* OR thyrotrophin* OR thyrotropin OR hypothyroid* OR hyperthyroid* OR thyroid dysfunction* OR triiodothyronin* OR thyroxin* OR TSH OR T4 OR T3 OR thyroid failure* OR thyroid disease* OR steroid* OR corticosteroid* OR corticoid* OR cortisone OR cortisol OR estrogen* OR oestrogen* OR androgen* OR testosterone* OR gonadotropin* OR estradiol* OR oestradiol*):ab,ti) |
| *O6* Electrolytes |
| ((electrolyt* OR sodium OR potassium OR kalium OR calcium OR phosphor* OR magnesium OR natrium OR osteoporo* OR osteopenia* OR ((bone OR osseous) NEAR/3 (densit* OR mineral* OR loss)) OR hyponatraemi* OR hyponatriaemi* OR hyponatremi* OR hyponatriemi* OR hypernatraemi* OR hypernatriaemi* OR hypernatriemi* OR hypernatriemi* OR hypokalaemi* OR hypokaliaemi* OR hypokaliemi* OR hypokalemi* OR hypercalcemi* OR hypercalcaemi* OR hypocalcemi* OR hypocalcaemi* OR hyperphosphatemia* OR hyperphosphataemi* OR hypophosphataemi* OR hypophosphatemia* OR hypomagnesemia* OR hypomagnesaemia*):ab,ti) |
| *O7* Red/white blood cells, hemoglobin |
| ((blood OR serum OR red NEXT cell* OR erythrocyte* OR leucocyte* OR lymphocyte* OR monocyte* OR neutrophil* OR basophil* OR eosinophil* OR haematocrit OR hematocrit OR transferrin OR transferrins OR apoferritin* OR ferritin* OR erythropoieti* OR hematopoie* OR hemopoie* OR haematopoie* OR haemopoie* OR hepcidin* OR haptoglobin* OR hemoglobin* OR haemoglobin* OR hemoglobulin* OR haemoglobulin* OR ferrohaemoglobin* OR ferrohemoglobin* OR iron):ab,ti) |
| *O8* Coagulation |
| ((fibrinogen* OR fibrinoly* OR ((blood) NEAR/3 (clotting OR clot OR clots OR coagulat*)) OR clotting NEXT time* OR coagulation NEXT factor* OR 'coagulation time' OR 'coagulation cascade' OR thrombin NEXT time* OR platelets OR INR OR prothrombi* OR thrombinogen* OR thrombogen* OR 'partial thromboplastin time' OR thrombinogen* OR hemostat* OR haemosta* OR 'vitamin K'):ab,ti) |
| *O9* Vitamin D |
| (('vitamin D' OR '25 hydroxyvitamin D' OR '25 OH' OR (25 NEXT hydroxy*) OR calcidiol OR calcitriol):ab,ti) |
| *O10* Liver function |
| ((((liver OR hepatic OR hepatocyte OR hepatocellular) NEAR/3 (function* OR inflammat*)) OR 'aspartate aminotransferase' OR 'aspartate transaminase' OR 'alanine aminotransferase' OR 'alanine transaminase' OR 'alkaline phosphatase' OR 'gamma glutamyltransferase' OR 'gamma glutamyl transferase' OR 'gamma glutamyl transpeptidase' OR 'gamma glutamyltranspeptidase' OR albumin*):ab,ti) |
| *O11* Biomarkers |
| ((marker OR markers OR biomarker*):ab,ti) |

**Table S1. Summary of study characteristics**

| **Author, year of publication** | **Study Location** | **SCI population** | | | | | | | | | | **Control population** | | | | | | **Assay type** | **Matching variable (i.e., age, sex, location, etc.)** | **Biomarkers measured** |
| --- | --- | --- | --- | --- | --- | --- | --- | --- | --- | --- | --- | --- | --- | --- | --- | --- | --- | --- | --- | --- |
|  |  | **No. of individuals** | **Health status** | **Number and % of males** | **Age**  **(mean y±SD )** | **SCI ethology** | **Percent of SCI individuals with complete SCI** | **SCI injury duration, (mean y±SD )** | **Injury level and comparison between the groups**  **Yes/no** | **BMI**  **(mean y±SD )** | **Medication use**  **Available/N.A.** | **No. of individuals** | **Health status** | **Number and % of males** | **Age**  **(mean y±SD )** | **BMI**  **(mean y±SD )** | **Medication use**  **Available/N.A.** |  |  |  |
| Akbal et al., 2014){Akbal, 2014 #2} | Turkey | 85 | healthy | 67 (78.8%) | 34.03(12.52) | - | 43 (51.82%) | 44 (  31.54) months | PP 60  TP 25  yes | 25.37 (4.24) | none | 38 | Healthy age-gender match | 75% | 35.54 (13.53) | 25.01 (3.85) | none | Hexokinase, glycerophosphate oxidase spectrophotometric method | P-wave, QT dispersion | Glucose, HDL-C, Triglycerides |
| (Akbal, Kurtaran, Selcuk, & Akyuz, 2013) | Turkey | 56 | healthy | n.a | 32.13 (11.82) | traumatic | 36 (64.28%) | 36.72 (30.33) months | PP 43  TP13  no | 25.02 (4.12) | none | 37 | Healthy age-gender match. Check up subject | n.a | 33.5 (7.0) | 26.7±3.7 | none | Immunosorbent, IFCC and enzyme immunoassay | H-FABP | Glucose, TC, HDL, TG, AST, ALT, LDH, Troponin, CK, |
| Arija-Balzquez 2013 | Spain | 10 | Healthy | 10 (100%) | 39.4 (12.3) | traumatic | 100% (10) | <8 weeks | T1-T12 | 25.1 (3.6) | None | 10 | Healthy | 100% | 36.7 (8.9) | 23.3 (3.3) | N.A. | Microparticle ELISA | Yes (age) | Testosterone, cortisol, osteocalcin |
| Bartoletti 2009 | Italy | 113 | Excluded DM | 113 (100%) | 61.3 (6.2) |  | 50.4% (57) | - | No (T12 above 91, | - | N.A. | 109 | Excluded DM | 109 (100%) | 65.4 (4.7) |  | N.A. | Microparticle ELISA | Yes (age) | Testosterone, PSA |
| (Bauman, Spungen, Wang, Pierson, & Schwartz, 2006) | USA | 10 | Healthy | 10 (100%) | 41 (9) | N.A | 10 (100%) | 16 **(9)** | PP 8  TP 2  No | 23.9 (4) | none | 10 | Healthy SCI twins | 10 (100%) | 41 (9) | 26.3 (3.5) | none | Radioimmune, autoanalyzer, colorimetric assay, Immunoradiometric | measures of adiposity (fat mass, %fat, BMI, weight), estradiol | Total T, Free T, FSH, LH, Albumin, IGF-I, hGH, estradiol, estrone, estrone sulfate, SHBG, |
| (Bauman, Spungen, Zhong, & Mobbs, 1996) | USA | 34 | helathy | 34 (100%) | 50 (11.66) | N.A | N.A | 11 (11.66) | PP 21  TP 13 | N.A | N.A | 48 | Healthy | 48 (100% | 41 (13.86) | N.A | N.A | RIA | No matching | Leptin |
| (Bauman & Spungen, 1994) | USA | 100 | Healthy | 100% male | 49 (14.14) | N.A | N.A | 18 (14.14) | PP 50  TP 50 | 25.15 (5.16) | N.A | 50 | Healthy Veteran controls | 100% male | 51 (14.14) | 26.9 (4.24) | N.A | RIA | Yes (age) | Insulin, glucose |
| (Bauman, Zhong, & Schwartz, 1995) | USA | 100 | Healthy | N.A | 51 (14) | N.A | N.A | 20 (13) | PP 49  TP 51 | 24.6 (4.5) | N.A | 50 | Healthy Veteran controls | N.A | 51 (13) | 26.7 (4.4) | N.A | Immunoradiometric, competitive protein binding, radioreceptor | yes | Calcium, Phosphate, albumin, alkaline phosphate, PTH, Vitamin D, |
| (Buchholz, McGillivray, & Pencharz, 2003) | CANADA | 28 | Healthy | 17 (61%) | 33.9 ± 9.2 | various | 18 (64%) | 11.4 ± 9.5 | none | 24.3 ± 6.0 | N.A | 34 | Healthy Students and staff of the university hospital | 24 (71%) | 29.1 ± 7.6 | 23.5 ± 1.8 | N.A | Intraassay, immunoassay | BMI | TSH, T3, T4, metanephrine |
| (Burr, Chem, & Nuseibeh, 1993) | UK | 39 | Non-healthy | 39 (100%) | 39 (12) | N.A | 27 (70%) | 6.38 (4.10) | CV 11  TH 11  LB 7 | N.A | N.A | 20 | Healthy | 20 (100%) | 37 (13) | N.A | N.A | ASTRA | No matching | Cr, Sodium, potassium, chloride, calcium, magnesium, phosphate, bicarbonate, citrate, albumin, urate, alkaline phosphatase, creatinine |
| (Campagnolo, Bartlett, Chatterton, & Keller, 1999) | USA | 18 | Healthy | 11 (61%) | 33 (10) | N.A | 18 (100%) | 68 (57)  months | PP 8  TP 10 | N.A | none | 18 | Healthy gender and age matched | 11 (61%) | 33 (10) | N.A | none | RIA, enzyme-linked immunosorbent, Enzyme immunoassay | Yes | Cortisol, DHEA, Dehydroepiandrosterone, ACTH, Prolactin |
| (Celik et al., 2007) | Turkey | 44 | Healthy | 44 (100%) | 34.6 (15.4) | traumatic | 23 (52%) | 18.6 (29.6)  months | PP 30  TP 14 | 23.8 (3.8) | N.A | 42 | Healthy age matched | 42 (100%) | 32.7 (8.2) | 25.2( 3.1) | N.A | Immuno, Radioimmuno assay | Yes (age) 12 months before and after injury | LH, Testosterone |
| (Cheville & Kirshblum, 1995) | USA | 30 | Healthy | 29 (97%) | 56 (15) | N.A | 21 (70%) | 25 (12) | PP 16  TP 14 | N.A | N.A | 30 | Healthy gender and age matched | 29 (97%) | 56 (15) | N.A | N.A | Fluorescence Polarization Immunoassay  Microparticle Enzyme  Immunoassay | Yes | TSH, T3, T4, T3RU, FTI |
| (Davies, Hayes, & Dekaban, 2007) | UK | 56 | Post-acute and chronic SCI | 42 (75%) | 40.6 (11.9.) | traumatic | 14 (25%) | 128 (99) months | PP 25  TP 31 | N.A | none | 35 | Healthy, Student population, hospital staff and community volunteers | 18 (51%) | 35.1 (9.8) | N.A | N.A | ELISA | Yes | RB, WBC, neutrophils, lymphocytes, monocytes, eosinophils, basophils, albumin, Hb, |
| (Dearwater et al., 1986) | USA | 94 | Healthy | 94 (100%) | 29 (10) | N.A | N.A | N.A | PP  TP | 2.17 (0.32) | yes | 126 | Healthy age match, students | 126 (100%) | 29 (5) | 2.58 (0.8) | N.A | RIA, enzyme, oxidase | Yes (age) | HDL, TC, TG, Insulin, Glucose |
| (Demirel, Demirel, Tukek, Erk, & Yilmaz, 2001) | Turkey | 69 | Rehabilitation period | 53 (77%) | 33.9 (11.37) | Traumatic, infection, vascular | 31 (45%) | 12.8 (13.45) | PP 45  TT 24 | 25.5 (4) | n.a | 52 | Young medical staff from annual check up | 38 (73%) | 35.5 (7.72) | 24.97 (3.3) | n.a | Enzymatic, automated blood analysis | Cardiovascular risk and contributing factors | Glucose, uric acid, TC, TG< HDL, LDL, VLDL |
| (Edwards, Bugaresti, & Buchholz, 2008) | Canada | 15 | Healthy | 12 (80%) | 39.8 (7.4) | traumatic | 11 (73%) | 16.5 (8.7) | PP 6 TP 9 | 25.2 (6.5) | yes | 16 | Community members | 12 (75%) | 38. 1 (8.4) | 25.5 (5.8) | Yes | Immunosorbent immunometric, intraassay, intraassay , | Yes | Glucose, Insulin, HOMA, CRP, TG, TC, HDL, LDL, OXI LDL, Adiponectin |
| (Fatima, Sharma, & Verma, 2016) | India | 22 | Acute CSCI | 22 (100%) | 35. 7(9.9) | N.A | N.A | 2 weeks | N.A | N.A | none | 22 | Healthy, relatives and community | 22 (100%) | 32.9 (10.3) | N.A | none | Enzyme-linked immunosorbent assay | No matching | Cortisol, melatonin |
| (Finsen, Indredavik, & Fougner, 1992) | Norway | 19 | mixed | 19 (100%) | 30 (13) | traumatic | N.A | 10. 4 (8.8) | CV 8  TH 4  LB 7 | N.A | yes | 19 | Healthy, nurses, medical students and blood donors | 19 (100%) | 30 (13) | N.A | none | Radioimmuno assay | yes | Hb, erythrocytes, Cr, alanine aminotransferase, alkaline phosphatase, Ca, phosphate, albumin, PTH, luteinizing hormone, FSH, Testosterone, GSH, free androgen |
| (Formisano et al., 1998) | Italy | 18 | Post-acute | 17 (95%) | 35 (10.4) | N.A | 15 (83%) | 3.98 | CV 5  TH 11  LB 2 | N.A | N.A | 10 | healthy | 5 (50%) | 32.7 | N.A | N.A | Immunofluorescence, cytometry  RIA or ELISA | Post comatose patients  No matching | ACTH, Cortisol, FSH, LH, PRL, T3, T4 |
| (Frost, Roach, Kushner, & Schreiber, 2005) | USA | 34 | Healthy | 34 (100%) | 43.09 (11.90) | N.A | N.A | 10.79 (8.18) | PP 18  TP 16 | N.A | N.A | 10 | Healthy, physicians | 10 (100%) | 30.10 (2.44) | N.A | N.A | ELISA | No matching | CRP, albumin, Cr, Hb, WBC, Hematocrit, TP |
| Gaspar 2014 | Brazil | 29 | Healthy | 29 (100%) | 32.7 (6.9) | traumatic | - | 7.64 (5.8) | T2-T12 | 23.7 (3.3) | N.A. | 16 | Healthy age and gender matched | 17 (100%) | 31.9 (5.8) | 26.8 (3.4) | N.A. | RIA, ELISA, intra-assay, CLIA | Yes (Age) | Testosterone, LH, FSH, Vitamin D, SHBG |
| (Gokkaya, 2013) | Turkey | 25 | healthy | 25 (100%) | 45 (7.48) | N.A | 25 (100%) | 124.5 (96.2) months | CV 2  TH 14  LB 9 | N.A | none | 32 | Outpatient urology unit | 32 (100%) | 44 (7) | N.A | none | immunoassay | Yes | Testosterone, FSH, LH |
| (Hjeltnes, De Groot, Birkeland, Falch, & Iversen, 2005) | Norway | 6 | Healthy | 6 (100%) | 36.1 (8.82) | N.A | 6 (100%) | More than 5 years | none | 24. 5(1.71) | N.A | 8 | Healthy age matched | 8 (100%) | 32.8 (4.24) | 22.9 (2.26) | N.A | RIA | yes | HDL, TG, Insulin, Glucose, Leptin |
| (Huang, Wang, & Lien, 1995) | Taiwan | 32 | Healthy | 32 (100%) | 34 (6) | traumatic | 32 (100%) | 7.7 (3.7) | CV 8  TH 24  PP24  TP 8 | 19.8 (3.2) | yes | 26 | Healthy, age matched | 26 (100%) | 34 (6) | 21.9 (2.4) | none | RIA | Yes. Time interval, Basal at 2 timepoints (-15 min and 0 min before intervention) | T3, T4, TSH, GH, Prolactin, Cortisol, FSH, LH, Testosterone, IGF-1 |
| Huang et al, 1998 | Taiwan | 25 | Healthy | 25, 100% | 35.4 | N.A | N.A | 7.5 |  | N.A | available | 25 | Healthy, age matched | 25, 100% | 37.1 | N. A | N. A | RIA | yes | Triiodothyronine, thyroxine, Testosterone,  Thyroid stimulating hormone,  Growth hormone, Prolactin, luteinizing hormone (LH),  Cortisol, Follicle stimulating hormone (FSH), Adrenocorticotropin |
| Hvarness et al, 2007 | Denmark | 31 | Healthy | 31. 100% | 49.8 ± 6.6 | N. A | 25.8 | 28.5 ± 8.8 | no | N. A | N. A | 31 | Healthy | 31, 100% | 50 ± 6.7 | N.A | N.A | N.A | yes | sex hormone-binding globulin (SHBG), Testosterone, Free androgen index (FAI) |
| Ibrahim et al, 2015 | USA | 30 | Healthy | 30, 100% | 36.5 ± 9.3 | N.A | N.A | N.A | no | N.A | N.A | 15 | Healthy, age matched | 15, 100% | 36.2 ± 10.5 | N.A | N.A | enzymatically amplified two-site immunoassay | yes | LH, FSH, Testosterone |
| (Invernizzi et al., 2015) | Italy | 28 | Healthy | 23 (82%) | 40.5 (7.1) | N.A | 22 (79%) | 7.57 (4.43) | PP 24  TP 4  Complete/incomplete | 24. 7 (3.4) | none | 15 | Healthy No intense physical exercise in 24h before phlebot. | 5 (33%) | 28.4 (4.1) | 22.8 (2.1) | none | ELISA. Only myostatin and sclerostin measured by ELISA. The biomarkers of interest were measured "in the laboratory analysis of our Hospital". | Complete/incomplete | Vitamin D, Phosphate, PTH, CRP, Calcium creatinine, IGF-1, BALP.  Note: eGFR (glom. filtration rate) is not a molecular biomarker. It is in the data extraction table however. |
| (Iversen et al., 2002) | Norway | 6 | Healthy | 6 (100%) | 36 (8.08) | traumatic | 6 (100%) | 11.5 (5.63) | C5-C7, complete | 22.9 (1.96) | yes | 8 | Healthy, | 8 (100%) | 33 **(4.24)** | 24.5 **(1.70)** | none | enzyme linked immunosorbent assay (CRP) Hb, WBC, INR, ESR, CT, HDL, LDL, TG measured using "standard techniques" | none | Hb, WBC, platelets, INR, ESR, CRP, CT, HDL, LDL, TG |
| (Jeon, Harber, & Steadward, 2003) | Canada | 6 | Healthy | 6 (100%) | 37 (7.35) | N.A | 6 (100%) | N.A | none | 25.9 (3.67) | N.A | 6 | Healthy, matched age, weight, height, BMI, and WC | 6 (100%) | 35 (8.57) | 29.1 (4.65) | N.A | RIA (leptin, insulin, GH) enzymatic w. auto analyzer (glucose), HPLC (epinephrine and norepinephrine) | Yes. Time interval | Leptin, Glucose, Insulin, GH, EPL |
| (Jones & Legge, 2019) | New Zealand | 20 | Healthy | 20 (100% | 33 (8.94) | traumatic | 9 (45%) | 10. 3(8.05) | PP 7  TP 13 | 23.5 (4.47) | N.A | 20 | Healthy advertisement found volunteers  age, height, weight matched | 20 (100% | 33 (8.94) | 24.1 (1.79) | N.A | Radioimmunoassay adiponectin, leptin, insulin). Autoanalyzer hexokinase (glucose) | yes | Leptin, Adiponectin, Fasting insulin, Fasting glucose, HOMA |
| (Jones, Legge, & Goulding, 2004) | New Zealand | 20 | Healthy | 20 (100% | 33 (8.94) | traumatic | N.A | 10. 3(8.05) | PP 9  TP 11 |  | none | 20 | Healthy age, weight, height | 20 (100%) | 33 (8.94) |  | none | RIA, enzymatic | yes | Glucose, Insulin, TC, HDL |
| (Kaya, Ünsal, Ordu Gökkaya, Aybay, & Özel, 2004) | Turkey | 75 | Healthy | 54 (72%) | 33.01 (9.28) | Traumatic | 54 (72%) | N.A | TP 22  PP 53 | N.A | None | 39 | Healthy | 26 (66.7%) | 35.69 (11.11) | N.A | None | immunometric assay | BMD | PTH, T3, T4, TSH, Ca, P, Alk P, Calcitonin, |
| Kikuchi et al, 1976 | USA | 15 | Healthy | 15, 100% | N.A | N.A | N.A | N.A | no | N.A | N.A | 15 | Healthy | 15, 100% | N.A | N.A | N.A | RIA | yes | LH, FSH, Testosterone |
| Kostovski et al, 2008 | Norway | 6 | Healthy | 6, 100% | 36±3.3 | Acute trauma | 100% | >5 | no | 22.9±0.8 | Available but not used during observation period | 8 | Healthy | 8, 100% | 33±  1.5 | 24.5±0.7 | N.A | RIA | no | SHBG, FAI |

| **Author, year of publication** | **Study Location** | **SCI population** | | | | | | | | | | **Control population** | | | | | | **Provided information on control group** | **Assay type** | **Matching variable (i.e., age, sex, location, etc.)** | **Outcomes measured** |
| --- | --- | --- | --- | --- | --- | --- | --- | --- | --- | --- | --- | --- | --- | --- | --- | --- | --- | --- | --- | --- | --- |
|  |  | No. of individuals | Health status | Number and % of males | Age  (mean y±SD) | SCI ethology | Percent of SCI individuals with complete SCI | SCI injury duration, (mean y±SD) | Injury level and comparison between the groups  Yes/no | BMI  (mean y±SD) | Medication use  Available/N.A. | No. of individuals | Health status | Number and % of males | Age  (mean y±SD) | BMI  (mean y±SD) | Medication use  Available/N.A. |  |  |  |  |
| La Favor 2011 | US | 14 | Healthy | 71.4% (10) | 34.8 (11.5) | Traumatic | unknown | 11 (9) | Mix | 24.1 (4.0) | none | 13 | Unknown | 69.2% (9) | 34.0 (10.5) | 25.1 (3.5) | excluded | Community | CRP turbidimetric assay, insulin electro immune assay, | Age, sex, height, body weight, race | Lipids, glucose |
| Li 2019 | US | 22 | Healthy | 0% (-) | 42.38 (10.92) | Unknown | 68.8 (15) | 14.0 (13.1) | Mix | 25.79 (6.9) | Excluded | 20 | Healthy | 0 (-) | 43.6 (11.9) | 26.7 (6.5) | Excluded | Community | Routine laboratory exam | Age, race, BMI | Glucose |
| Liang 2007 | US | 185 | Mixed | 100% (185) | 39.2 (10.4) | Unknown | 55.7 (103) | 11.7 (1-40.4 y) | Mix | 26.1 (6.0) | Obesity 35.1%, hypertension 44.9%, increased fasting glucose 23.8% | 185 | Mixed | 100 (185) | 39.1 (10.4) | 27.1 (5.1) | Obesity 28.6%, hypertension 37.8%, increased fasting glucose 36.2% | Community | Routine laboratory exam | Age, race | Lipids, glucose |
| Lieberman 2014 | US | 100 | Unknown | 78% (78) | 45.3 (5.1) | Mixed | 66 (103) | >1 year | Mix | 26.8 (7.2) | Unknown | 100 | Unknown | 78 | 44.8 (4.4) | 27.8 (4.3) | Unknown | Community | Routine laboratory exam | Age, race | Lipids, glucose, inflammatory markers |
| Maimoun 2005 | France | 7 | Healthy | 100 (7) | 31.3 (9.5) | Unknown | Unknown | Unknown | Mix | 21.5 (2.3) | N.A | 12 | Healthy | 100 (12) | 26.9 (4.2) | 22.6 (1.9) | N.A | Unknown | Enzyme-linked immunosorbent assays (ELISAs) | Age | Endocrine |
| Maruyama 2008 | Japan | 45 | Mixed | 100% (45) | 57 (8) | Mixed | 62.2 (28) | 26 (0.5 – 57) | Mix | 24.1 (4.8) | Antihypertensives 30%, Antihyperlipidemics 18% | 125 | Unknown | 100% (125) | 57 (9) | 23.1 (3.0) | Unknown | Unknown | Routine laboratory exam | No matching | Glucose, lipids |
| Mathian et al, 1999 | France | 13 | Healthy | 13, 100% | 32.3 ± 10.1 | Unknown | N.A | N.A | No | N.A | N.A | 7 | Healthy | 7,100% | 29.7 ±2.4 | N.A | N.A |  | Radioimmunoassay | Yes (Age) | Free testosterone (FT), testosterone, FSH |
| Matos Souza 2011 | Brazil | 34 | Healthy | 100% (34) | 31.9 (3) | Unknown | 52.9 (18) | 6.7 (0.8) | Mix | 23.6 (0.7) | N.A | 31 | Healthy | 100% (31) | 31.1 (3) | 24.5 (0.7) | N.A | Hospital staff | Enzyme-linked immunosorbent assays (ELISAs) | No matching | Lipids, glucose, Inflammatory markers |
| Mojtahedi 2008 (Mojtahedi, Valentine, Arngrimsson, Wilund, & Evans, 2008) | US | 14 | Athletes | 50% (7) | 22.5 (3.7) | Unknown | Unknown | 16.5 (5.7) | Paraplegia | 22.2 (3.6) | Unknown | 17 | Unknown | 47.8 (8) | 22.5 (2.7 | 24.3 (2.7) | Unknown | Community (sedentary) | Routine laboratory exam | Age, BMI | Glucose, lipids |
| Munakata 1997 | Japan | 19 | Healthy | Unknown | Unknown | Unknown | Unknown | Unknown | Unknown | Unknown | N.A | 16 | 43.8 (7) | 42 (3) | Unknown | Unknown | Unknown | Unknown | High-performance liquid chromatography and immunoradiometric assay kit | No matching | Endocrine (Epinephrine, norepinephrine, atrial natriuretic peptide) |
| Naderi et al, 2003 | Iran | 55 | Healthy | 55, 100% | 37 ± 1·8 | Traumatic injury | 53% | 16 ± 0·8 | No | 18·8 ± 2·2 | Available | 70 | Healthy | 70, 100% | 34 ± 1·2 | 22·3 ± 2·1 | N.A |  | Radioimmunoassay | Yes (Age) | LH, FSH, Testosterone |
| Nance et al, 1985 | Canada | 10 | Healthy | 10. 100% | 30.6 ± 14 | NA | NA | NA | No | N.A | N.A | 3 | Healthy | 3, 100% | 34.3 ± 13.8 | N.A | N.A |  | Radioimmunoassay | Yes (Age) | LH, FSH, Testosterone |
| Nelson 2007 | US | 20 | Healthy | 55% (11) | 16.9 (3) | Traumatic | Unknown | 4.8 (4.0) | Paraplegia | Unknown | N.A | 60 | Healthy | 45% (27) | 16.2 (2.5) | Unknown | N.A | Community (obesity clinic) | Routine laboratory exam | No matching | Glucose, lipids |
| Paim 2013 | Brazil | 42 | Healthy | 100% (42) | 33.18 (1.5) | Unknown | Unknown | 8.4 (0.98) | Mix | 23 (0.79) | Not applicable | 16 | Healthy | 100% (16) | 29.6 (1.3) | 25.1 (0.8) | Not applicable | Unknown | Routine laboratory exam | No matching | Lipids, glucose |
| Radulovic 2015 | US | 12 | Healthy | 83.3 (10) | 48 (11) | Unknown | Unknown | 21 (14) | Tetraplegia | 24.3 (3.4) | N.A | 10 | Unknown | 100 (10) | 48 (10) | 27.6 (50) | Unknown | Unknown | Commercial radioimmunoassay kits | Age, BMI | Inflammatory markers |
| Safarinejad 2001 | Iran | 76 | Healthy | 100% (76) | 38.8 (31-47) | Traumatic | 31% (24) | 16 (13-20) | Mix | Unknown | N.A | 41 | Healthy | 100% (41) | 34.7 (27-42) | Unknown | N.A | Unknown (Normospermic controls) | Commercial radioimmunoassay kits | No matching | Endocrine (LH, FSH, testosterone, prolactin) |
| Schreiber 2017 | Brazil | 22 | Healthy | 100% (22) | 34.3 (8.6) | Unknown | 100% (22) | 7.1 (4.1) | Mix | 22.7 (2.5) | N.A | 11 | Unknown | 100% (11) | 28.9 (4.5) | 26.1 (2.8) | Unknown | Unknown | Routine laboratory exam | No matching | Lipids, glucose, inflammatory markers |
| Shin et al, 2004(Shin, 2004) | Korea | 67 | Healthy | 67, 100% | 35.1±10.7 | Traumatic, non-traumatic | 61% | 10.9±26.1 | No | N.A | Available | 59 | Healthy | 59, 100% | 32.2 ± 4.1 | NA | NA |  | Radioimmunoassay | No | FSH, LH, Testosterone |
| Sica 1993 | US | 13 | Healthy | 100% (13) | 49.76 (10.1) | Unknown | 100% | 6 months | Mix | Unknown | N.A | 6 | Healthy | 100% (6) | Unknown | Unknown | Unknown | Community | Double antibody | No matching | Endocrine (Atrial natriuretic peptide) |
| Tsitouras et al, 1995 | USA | 20 | Healthy | 20, 100% | 42 ± 8.9 | N.A | N.A | 15 ±8.9 | No | 26.9 ± 4 | N.A | 16 | Healthy | 16, 100% | 39 ± 16 | 24.5 ±2.8 | N.A |  | Radioimmunoassay | Yes (Age) | TT, FT, FSH, LH |
| Vaziri 1994 | US | 40 | Healthy | 100% (40) | 24-69 | Unknown | Unknown | 3-50 | Mix | Unknown | N.A | 14 | Healthy | 100% (14) | 28-67 | Unknown | N.A | Unknown | High performance liquid chromatography | No matching | Endocrine, vitamin (Vitamin D, parathormone) |
| Wang 2007 | Taiwan | 89 | Healthy | 100% (89) | 39.3 (1.1) | Unknown | 100% | 10.8 (0.7) | Mix | 22.3 (0.6) | N.A | 37 | Healthy | 100% (37) | 38.1 (1.4) | 23.3 (0.6) | N.A | Unknown | Enzyme-linked immunosorbent assays (ELISAs) | Age, BMI | Inflammatory markers, lipids, glucose |
| Zhang 2018 | China | 20 | Healthy | 100% (40) | 39.9 (10.57) | Unknown | 100% | Unknown | Tetraplegia | 23.11 (2.876) | N.A | 23 | Healthy | 100% (23) | 40 (9.03) | 24.8 (2.677) | N.A | Unknown | Routine laboratory exam | No matching | Lipids, glucose. electrolytes |
| Zhou 1993 | US | 92 | Healthy | 100% (92) | 48 (15) | Unknown | Unknown | 1-46 | Mix | Unknown | N.A | 28 | Healthy | 100% (28) | 32 (11) | Unknown | N.A | Unknown | Liquid scintillation counter | No matching | Electrolytes, Vitamins (Vitamin D) |

## TC, total cholesterol; HDL, high density lipoprotein; LDL, low-density lipoprotein; HOMA-IR, Homeostatic Model Assessment for Insulin Resistance; CRP, c-reactive protein; hsCRP, highly sensitive c-reactive protein; IL-6, interleukin 6; TNF alpha, tumor necrosis factor alpha; TSH, thyroid stimulating hormone; T3, triiodothryoxine; T4 tetraiodothyroxine; LH, luteinizing hormone; FSH, follicle stimulating hormone; GH, growth hormone; ACTH, adrenocorticotropic hormone; IGF-1, insulin-like growth factor-1; PTH, parathyroid hormone; RBC, red blood cells; WBC, white blood cells; HB, hemoglobin; ESR, erythrocyte sedimentation rate

**Table S2. Characteristics of studies excluded from meta-analysis**

| **Author, publication year** | **Findings** | **Reason for exclusion in meta-analysis** |  |
| --- | --- | --- | --- |
| Insulin | | | |
| (Duckworth et al., 1980) | Peripheral insulin activity was found to be significantly lower in glucose intolerant SCI individuals when compared to control group. | Unique outcome (Peripheral insulin activity). Only graphic available data. |  |
| (Karlsson, 1999) | Results showed decreased insulin sensitivity and increased fat tissue mass in SCI individuals. | Non- related outcome results (biomarkers measures from adipose tissue, not blood) |  |
| (La Fountaine et al., 2015) | The SCI group showed similar lipoprotein profile compared to ABI group, however, the SCI group lipids composition was associated with increased atherogenic risk and a higher tendency to present insulin resistance. | Use of similar cohort. |  |
| (La Fountaine, Cirnigliaro, Kirshblum, McKenna, & Bauman, 2017) | VAT volume was significantly higher in  SCI individuals when compared to ABI group. Also, higher levels of TG and lower levels of HDL were found for the SCI group. Insulin levels were not significantly different among all groups. | Use of similar cohort |  |
| Inflammatory Markers | | | |
| (Liang, Mojtahedi, Chen, & Braunschweig, 2008) | SCI had elevated CRP and decreased HDL compared with ABI. | Duplicate publication |  |
| (Matos-Souza et al., 2011) | SCI had elevated CRP and impaired left ventricular function compared to ABI. | Double publication |  |
| (Wang et al., 2009) | Lower cholesterol, TG, and HDL levels were found in SCI individuals compared to ABI group. | Same cohort |  |
| Hormones and Growth Factors | | | |
| (Bauman et al., 2001) | Plasma homocysteine concentration was found to be higher in SCI individuals than ABI. | No mean and SD or SEM values. Only graphic available data. |  |
| (Bauman & Spungen, 2001) | Results showed lower levels of total testosterone in SCI population compared to ABI population. | No mean and SD or SEM values |  |
| (Bauman, La Fountaine, Cirnigliaro, Kirshblum, & Spungen, 2018) | Higher levels of FSH rand LH response to GnRH, could lead to believe hypothalamic-pituitary dysfunction in individuals with SCI. | Use of similar cohort |  |
| (Bauman, La Fountaine, Cirnigliaro, Kirshblum, & Spungen, 2017) | Lower serum testosterone concentrations in SCI individuals than in AB individuals. | Use of similar cohort |  |
| (Brackett, Lynne, Weizman, Bloch, & Abae, 1994) | Sperm motility and per cent normal sperm morphology were decreased in SCI individuals compared to ABI. | Use of similar cohort |  |
| (Brackett, Lynne, Weizman, Bloch, & Padron, 1994) | No signs of generalized scrotal thermoregulatory dysfunction in SCI males. Higher gonadotropin levels were not associated with increased scrotal temperatures in SCI men. | No available SI unit for LH and FSH |  |
| (Cortes-Gallegos et al., 1982) | Plasma androgen levels were consistently lower during the first three months after SCI injury. Suggestion of association between time after SCI injury and plasma androgens concentrations. | No mean and SD or SEM values |  |
| (Jeon et al., 2003) | SCI group had significantly higher leptin levels than the ABI group. | Use of similar cohort |  |
| (Madduri, de Salvo, & Seebode, 1979) | No significant differences were found in plasma androgens including testosterone and estradiol, between SCI and ABI population. | No SD |  |
| (Maimoun et al., 2002) | PTH and vitamin D were decreased in individuals with SCI when compared to ABI. | Duplicate publication |  |
| (Maimoun et al., 2004) | Leptin levels were elevated in individuals with SCI when compared to ABI. | Duplicate publication |  |
| (Maimoun et al., 2006) | Total testosterone was significantly lower in the SCI patients, whereas FSH was significantly higher. | No mean and SD or SEM values. Only graphic available data. |  |
| (Schmid et al., 2000) | No significant differences in lipoprotein(a) were found between SCI individuals and ABI subjects. However, differences in serum catecholamine levels derived from impairment of sympathetic nervous system and VO2max levels were found in SCI individuals, related to the abnormal lipid profile and higher risk for cardiovascular diseases. | Unique outcome (Adrenaline & Noradrenaline) |  |
| (Shetty, Sutton, Mattson, & Rudman, 1993) | Endocrine alterations in SCI individuals may contribute to the decrease in lean body mass and towards muscle atrophy. Moreover, the presence of hyposomatomedinemia could lead to a higher risk of pressure sores and osteoporosis. | Unique outcome (Somatomedin-C) |  |

## TG, Triglycerides; HDL, high density lipoprotein; CRP, c-reactive protein; LH, luteinizing hormone; FSH, follicle stimulating hormone; PTH, parathyroid hormone.

**Table S3. Subgroup analysis, Total Testosterone according to study characteristics**

| **Study Characteristics** | **Stratum** | **# Of studies** | **Weighted mean difference** | **95% confidence interval** | **I^2^ test for heterogeneity** | **p-value** |  |
| --- | --- | --- | --- | --- | --- | --- | --- |
| **Total Testosterone (nmol/l)** | | | | | | | |
| **Age** | **Below Median** | 8 | -2.413 | -4.942, 0.116 | 83.1% | 0.676 |  |
|  | **Above Median** | 9 | -3.423 | **-5.807, -1.039*** | 89.0% |  |  |
| **Type of Injury** | **Mixed** | 16 | -2.507 | **-4.547, -0.467*** | 90.8% | 0.989 |  |
|  | **Paraplegia** | 1 | -2.462 | -7.674, 2.751 | - |  |  |
|  | **Tetraplegia** | - | - | - | - |  |  |
| **Time since Injury** | **Below Median** | 6 | -0.508 | -2.631, 1.615 | 58.7% | 0.129 |  |
|  | **Above Median** | 7 | -3.009 | **-5.323, -0.696*** | 88.6% |  |  |
| **Participants (#)** | **Below Median** | 9 | -2.570 | -5.251, 0.111 | 89.8% | 0.999 |  |
|  | **Above Median** | 9 | -2.623 | -5.574, 0.328 | 90.3% |  |  |
| **Completeness of Injury** | **Complete** | 7 | -3.073 | -7.341, 1.196 | 91.2% | 0.751 |  |
|  | **Mix** | 8 | -3.61 | **-5.734, -1.487*** | 79.7% |  |  |
| **Location** | **Asia** | 7 | -1.125 | -3.351, 1.101 | 76.3% | 0.348 |  |
|  | **Europe** | 5 | -4.892 | -10.392, 0.608 | 94.6% |  |  |
|  | **North America** | 5 | -1.774 | -5.145, 1.596 | 88.8% |  |  |
|  | **South America** | 1 | -7.246 | -12.363, -2.129 | - |  |  |
| **Gender matching***^1^ | **Yes** | 16 | -2.479 | **-4.471,-0.486 *** | 90.9% | 0.668 |  |
|  | **No** | 2 | -4.100 | **-6.001, -2.199*** | - |  |  |
| **Age matching***^1^ | **Yes** | 16 | -2.479 | **-4.471, -0.486*** | 90.9% | 0.668 |  |
|  | **No** | 2 | -4.100 | **-6.001, -2.199*** | - |  |  |
| **Health Status** | **Healthy** | 17 | -2.853 | **-4.672, -1.033*** | 87.7% | 0.391 |  |
|  | **Non-healthy** | - |  |  |  |  |  |
|  | **Mixed** | 1 | 0.600 | -0.934, 2.134 | - |  |  |
| **Assay** | **ELISA** | **3** | -7.407 | **-12.838,-1.976*** | 80.5% | **0.044*** |  |
|  | **RIA** | **11** | -2.070 | -4.383, 0.243 | 88.4% |  |  |
|  | **Other** | **3** | -0.093 | -2.446, 2.260 | 70.3% |  |  |

*Indicates statistically significant result, pvalue < 0.05

*^1^ studies were adjusted for both, age and gender

**Table S4. Subgroup analysis, LH according to study characteristics**

| **Study Characteristics** | **Stratum** | **# Of studies** | **Weighted mean difference** | **95% confidence interval** | **I^2^ test for heterogeneity** | **p-value** |
| --- | --- | --- | --- | --- | --- | --- |
| **LH (IU/l)** | | | | | | |
| **Age** | **Below Median** | 7 | 1.431 | -0.532, 3.394 | 89.5% | 0.287 |
|  | **Above Median** | 7 | -0.320 | -1.493, 0.852 | 84.7% |  |
| **Type of Injury** | **Mixed** | 14 | 0.286 | -0.639, 1.211 | 86.6% | **0.000*** |
|  | **Paraplegia** | - |  |  |  |  |
|  | **Tetraplegia** | - |  |  |  |  |
| **Time since Injury** | **Below Median** | 6 | 1.720 | **0.053, 3.386*** | 71.7% | 0.145 |
|  | **Above Median** | 6 | -0.584 | -1.908, 0.739 | 93.1% |  |
| **Participants (#)** | **Below Median** | 7 | 0.594 | -0.405, 1.593 | 74.0% | 0.702 |
|  | **Above Median** | 8 | 0.127 | -1.356, 1.611 | 86.7% |  |
| **Completeness of Injury** | **Complete** | 5 | 0.701 | -0.467, 1.870 | 33.7% | 0.741 |
|  | **Mix** | 7 | 0.159 | -1.625, 1.942 | 92.9% |  |
| **Location** | **Asia** | 7 | -0.146 | -1.689, 1.397 | 86.3% | 0.551 |
|  | **Europe** | 2 | 12.541 | -10.985,36.066 | 92.7% |  |
|  | **North America** | 5 | 0.323 | -0.462, 1.108 | 29.7% |  |
|  | **South America** | 1 | 2.000 | **0.256, 3.744*** | - |  |
| **Gender matching***^1^ | **Yes** | 12 | 0.449 | -0.321, 1.219 | 77.6% | 0.569 |
|  | **No** | 3 | 1.481 | -3.052, 6.013 | 91.4% |  |
| **Age matching***^1^ | **Yes** | 12 | 0.449 | -0.321, 1.219 | 77.6% | 0.569 |
|  | **No** | 3 | 1.481 | -3.052, 6.013 | 91.4% |  |
| **Health Status** | **Healthy** | 13 | 0.022 | -0.930, 0.974 | 84.5% | **0.007*** |
|  | **Non-healthy** | 1 | 25.450 | **12.715, 38.185*** | - |  |
|  | **Mixed** | 1 | 1.380 | **0.703, 2.057*** | - |  |
| **Assay** | **ELISA** | - | - | - | - | 0.733 |
|  | **RIA** | 12 | 0.159 | -0.935, 1.254 | 88.0% |  |
|  | **Other** | 3 | 0.693 | -0.676, 2.062 | 67.5% |  |

LH, luteinizing hormone

*Indicates statistically significant result, pvalue < 0.05

*^1^ studies were adjusted for both, age and gender

**Table S5. Subgroup analysis, FSH according to study characteristics**

| **Study Characteristics** | **Stratum** | **# Of studies** | **Weighted mean difference** | **95% confidence interval** | **I^2^ test for heterogeneity** | **p-value** |
| --- | --- | --- | --- | --- | --- | --- |
| **FSH (IU/l)** | | | | | | |
| **Age** | **Below Median** | 6 | 1.277 | -0.548, 3.102 | 79.1% | 0.414 |
|  | **Above Median** | 7 | -0.040 | -3.157, 3.077 | 97.3% |  |
| **Type of Injury** | **Mixed** | 12 | 0.946 | -0.967, 2.860 | 95.9% | **0.000*** |
|  | **Paraplegia** | - | - | - | - |  |
|  | **Tetraplegia** | - | - | - | - |  |
| **Time since Injury** | **Below Median** | 5 | 1.947 | **0.758, 3.136*** | 48.1% | **0.035*** |
|  | **Above Median** | 5 | -1.979 | -5.680, 1.722 | 98.1% |  |
| **Participants (#)** | **Below Median** | 6 | 1.836 | **0.593, 3.080*** | 74.1% | 0.217 |
|  | **Above Median** | 7 | -0.364 | -2.798, 2.070 | 93.5% |  |
| **Completeness of Injury** | **Complete** | 5 | 2.193 | **0.737, 3.648*** | 28.1% | 0.122 |
|  | **Mix** | 6 | -0.934 | -4.303, 2.435 | 97.7% |  |
| **Location** | **Asia** | 6 | -1.001 | -3.249, 1.248 | 91.1% | 0.419 |
|  | **Europe** | 2 | 1.076 | **0.575, 1.577*** | 0.0% |  |
|  | **North America** | 4 | 2.691 | **1.584, 3.799*** | 11.6% |  |
|  | **South America** | 1 | 2.680 | **0.822, 4.538*** | - |  |
| **Gender matching***^1^ | **Yes** | 11 | 1.462 | -0.313, 3.236 | 93.8% | **0.015*** |
|  | **No** | 2 | -4.108 | **-4.932, -3.284*** | 0.0% |  |
| **Age matching***^1^ | **Yes** | 11 | 1.462 | -0.313, 3.236 | 93.8% | **0.015*** |
|  | **No** | 2 | -4.108 | **-4.932, -3.284*** | 0.0% |  |
| **Health Status** | **Healthy** | 12 | 0.602 | -1.686, 2.890 | 95.7% | 0.872 |
|  | **Non-healthy** | - | - | - | - |  |
|  | **Mixed** | 1 | 1.070 | **0.557, 1.583*** | - |  |
| **Assay** | **ELISA** | - | - | - | - | 0.298 |
|  | **RIA** | 10 | 0.095 | -2.050, 2.240 | 96.6% |  |
|  | **Other** | 3 | 2.369 | **0.992, 3.746*** | 0.0% |  |

FSH, follicle stimulating hormone

*Indicates statistically significant result, pvalue < 0.05

*^1^ studies were adjusted for both, age and gender

**Table S6. Subgroup analysis, Insulin according to study characteristics**

| **Study Characteristics** | **Stratum** | **# Of studies** | **Weighted mean difference** | **95% confidence interval** | **I^2^ test for heterogeneity** | **p-value** |  |
| --- | --- | --- | --- | --- | --- | --- | --- |
| **Insulin (pmol/l)** | | | | | | | |
| **Age** | **Below Median** | 6 | -0.332 | -7.493, 6.829 | 0.0% | 0.339 |  |
|  | **Above Median** | 7 | 6.251 | -3.306, 15.807 | 53.4% |  |  |
| **Type of Injury** | **Mixed** | 9 | 5.367 | -1.953, 12.687 | 51.7% | 0.457 |  |
|  | **Paraplegia** | 2 | 11.707 | -19.399, 42.813 | 0.0% |  |  |
|  | **Tetraplegia** | 1 | -6.200 | -17.576, 5.176 | - |  |  |
| **Time since Injury** | **Below Median** | 4 | 8.163 | -0.435, 16.762 | 46.2% | 0.786 |  |
|  | **Above Median** | 4 | 5.578 | -3.238, 14.394 | 1.5% |  |  |
| **Participants (#)** | **Below Median** | 7 | 1.009 | -6.173, 8.191 | 0.0% | 0.590 |  |
|  | **Above Median** | 6 | 4.983 | -5.951, 15.917 | 67.9% |  |  |
| **Completeness of Injury** | **Complete** | 3 | 4.704 | -13.773, 23.181 | 81.6% | 0.891 |  |
|  | **Mix** | 4 | 2.906 | -14.091, 19.903 | 53.4% |  |  |
| **Location** | **Asia** | 1 | 14.380 | **9.757, 19.003*** | - | **0.022*** |  |
|  | **Europe** | 1 | -6.200 | -17.576, 5.176 | - |  |  |
|  | **North America** | 9 | 1.856 | -5.419, 9.130 | 1.8% |  |  |
|  | **Oceania** | 2 | 6.210 | -5.565, 17.985 | 0.0% |  |  |
| **Gender matching***^1^ | **Yes** | 11 | 3.580 | -3.697, 10.858 | 58.0% | 0.680 |  |
|  | **No** | 2 | 11.707 | -19.399, 42.813 | 0.0% |  |  |
| **Age matching***^1^ | **Yes** | 12 | 3.510 | -3.631, 10.651 | 54.4% | 0.554 |  |
|  | **No** | 1 | 15.070 | -17.786, 47.926 | - |  |  |
| **Health Status** | **Healthy** | 11 | 5.659 | -0.817, 12.135 | 45.9% | **0.047*** |  |
|  | **Non-healthy** | - | - | - | - |  |  |
|  | **Mixed** | - | - | - | - |  |  |
| **Assay** | **ELISA** | 1 | 14.380 | **9.757, 19.003*** | **-** |  |  |
|  | **RIA** | 5 | 1.687 | -5.847, 9.221 | 0.0% |  |  |
|  | **Other** | 5 | 5.076 | -6.234, 16.386 | 35.0% |  |  |
| **Source of control** | **Community** | 7 | 4.418 | -2.439, 11.275 | 0.0% | 0.595 |  |

*Indicates statistically significant result, pvalue < 0.05

*^1^ studies were adjusted for both, age and gender

**Table S7. Glucose Subgroup Analysis According to Study Characteristics**

| **Study Characteristics** | **Stratum** | **# Of studies** | **Weighted mean difference** | **95% confidence interval** | **I^2^ test for heterogeneity** | **p-value** |
| --- | --- | --- | --- | --- | --- | --- |
| **Glucose (mmol/l)** | | | | | | |
| **Age** | **Below Median** | 11 | -0.156 | -0.316, 0.003 | 73.9% | 0.220 |
|  | **Above Median** | 11 | 0.007 | -0.167, 0.181 | 88.7% |  |
| **Type of Injury** | **Mixed** | 17 | -0.11 | -0.23, 0.01 | 85.3% | 0.050 |
|  | **Paraplegia** | 2 | -0.15 | -0.32, 0.02 | 0.0% |  |
|  | **Tetraplegia** | 2 | 0.74 | **0.24, 1.24*** | 0.0% |  |
| **Time since Injury** | **Below Median** | 8 | -0.176 | -0.370, 0.019 | 80.2% | 0.262 |
|  | **Above Median** | 8 | -0.012 | -0.203, 0.178 | 90.5% |  |
| **Participants (#)** | **Below Median** | 11 | -0.095 | -0.222, 0.031 | 12.4% | 0.959 |
|  | **Above Median** | 11 | -0.087 | -0.230, 0.057 | 90.9% |  |
| **Completeness of Injury** | **Complete** | 5 | 0.06 | -0.25, 0.36 | 67.0% | 0.716 |
|  | **Mix** | 10 | -0.01 | -0.21, 0.20 | 88.0% |  |
| **Location** | **Asia** | 5 | 0.23 | -0.07, 0.52 | 87.0% | 0.068 |
|  | **Europe** | 1 | 0.40 | -0.43, 1.23 | - |  |
|  | **North America** | 10 | -0.23 | **-0.36, -0.10*** | 62.7% |  |
|  | **South America** | 4 | -0.11 | -0.28, 0.06 | 28.1% |  |
|  | **Oceania** | 2 | -0.28 | **-0.48, -0.08*** | 0.0% |  |
| **Gender matching** | **Yes** | 16 | -0.112 | -0.236, 0.011 | 85.8% | 0.402 |
|  | **No** | 6 | 0.022 | -0.229, 0.273 | 65.2% |  |
| **Age matching** | **Yes** | 17 | -0.113 | -0.235, 0.009 | 84.8% | 0.376 |
|  | **No** | 5 | 0.039 | -0.235, 0.314 | 72.2% |  |
| **Health Status** | **Healthy** | 18 | -0.050 | -0.187, 0.088 | 79.7% | 0.532 |
|  | **Non-healthy** | - | - | - | - |  |
|  | **Mixed** | 2 | -0.212 | -0.495, 0.070 | 62.9% |  |
| **Source of Control** | **Community** | 9 | -0.238 | **-0.359, -0.118** | 69.6% | 0.478 |
|  | **Hospital** | 2 | 0.467 | **0.218, 0.716*** | 12.5% |  |
|  | **Mixed** | - | - | - | - |  |
| **Assay** | **Routine Laboratory Exams** | 7 | -0.119 | -0.342, 0.104 | 90.1% | **0.000*** |

*Indicates statistically significant result, pvalue < 0.05

**Table S8. Relationship of hormones**

|  | **β** | **95% CI** | **p-value** |
| --- | --- | --- | --- |
| **Spinal cord injury** | | | |
| FSH and LH | 0.594 | -0.006, 1.194 | 0.052 |
| Testosterone and LH | **1.104** | **0.097, 2.111** | **0.034*** |
| Testosterone and FSH | 0.242 | -0.866, 1.352 | 0.640 |
|  |  |  |  |
| **Able bodied individuals** | | | |
| FSH and LH | 0.075 | -0.678, 0.828 | 0.824 |
| Testosterone and LH | 0.958 | -0.507, 2.424 | 0.180 |
| Testosterone and FSH | -0.620 | -2.609, 1.369 | 0.507 |

## LH, luteinizing hormone; FSH, follicle stimulating hormone.

## *Indicates statistically significant result, pvalue < 0.05

**Table S9. Relationship of hormones with study characteristics**

|  | **β** | **95% CI** | **p-value** |
| --- | --- | --- | --- |
| **Testosterone** | | | |
| Injury duration | -0.170 | -0.398, 0.058 | 0.130 |
| Age | -0.189 | -0.389, 0.011 | 0.063 |
| Percent male | -0.026 | -0.099, 0.046 | 0.454 |
| Participants | **-0.040** | **-0.075, -0.005** | **0.027*** |
| **FSH** | | | |
| Injury duration | -0.486 | -0.990, 0.017 | 0.057 |
| Age | -0.124 | -0.510, 0.261 | 0.493 |
| Percent male | -0.041 | -0.083, 0.0001 | 0.051 |
| Participants | **-0.064** | **-0.100, -0.027** | **0.003*** |
| **LH** | | | |
| Injury duration | -0.486 | -0.990, 0.017 | 0.057 |
| Age | -0.124 | -0.510, 0.261 | 0.493 |
| Percent male | -0.041 | -0.083, 0.0001 | 0.051 |
| Participants | **-0.064** | **-0.100, -0.027** | **0.003*** |
| **Insulin** | | | |
| Injury duration | 0.261 | -1.738, 2.260 | 0.760 |
| Age | 0.127 | -0.922, 1.177 | 0.792 |
| Percent male | 0.106 | -0.611, 0.825 | 0.747 |
| Participants | -0.003 | -0.125, 0.119 | 0.956 |

## LH, luteinizing hormone; FSH, follicle stimulating hormone.

*Indicates statistically significant result, pvalue < 0.05

**Fig. S1 Publication Bias, Funnel Plots and Egger’s Test**


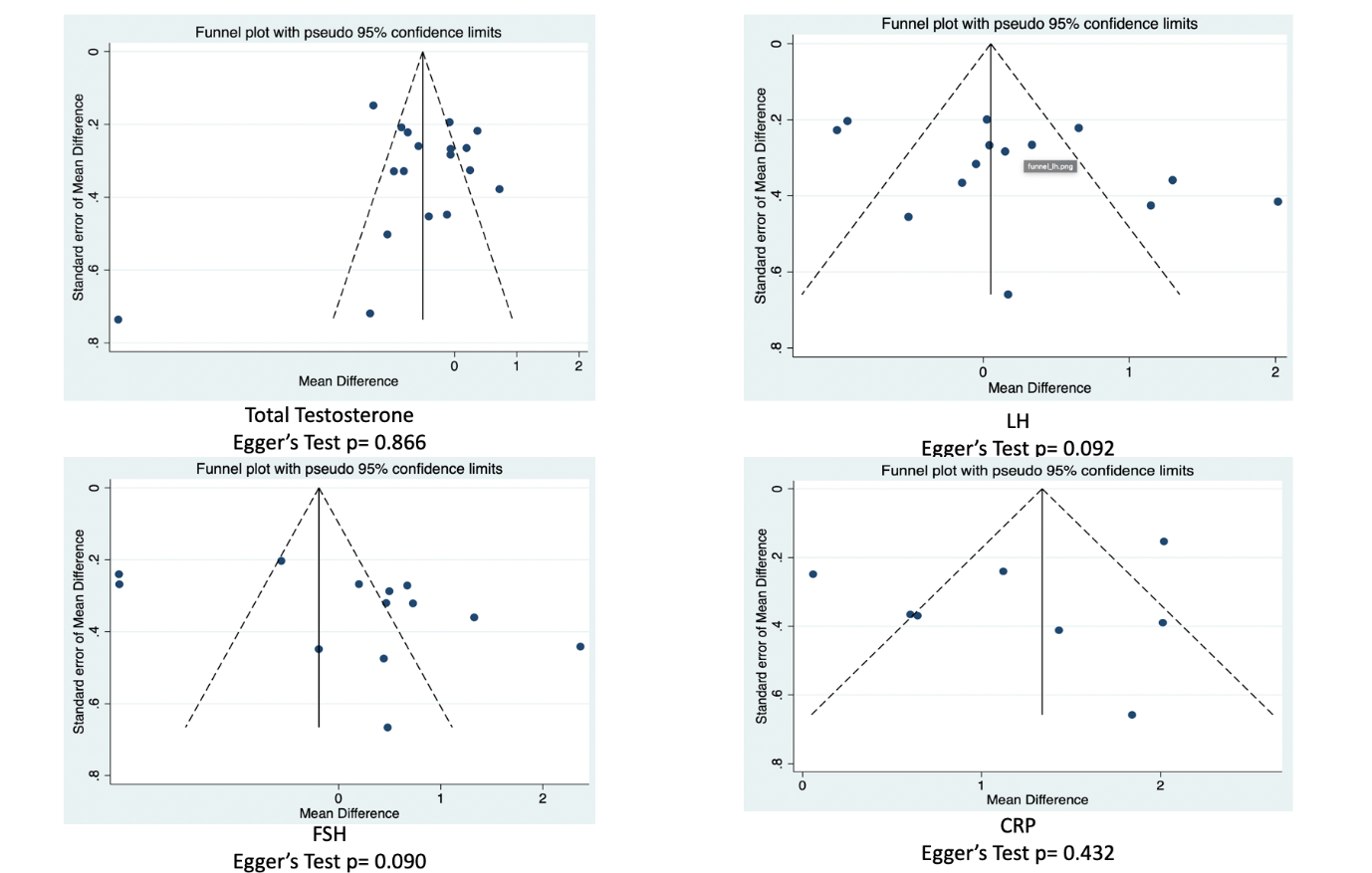


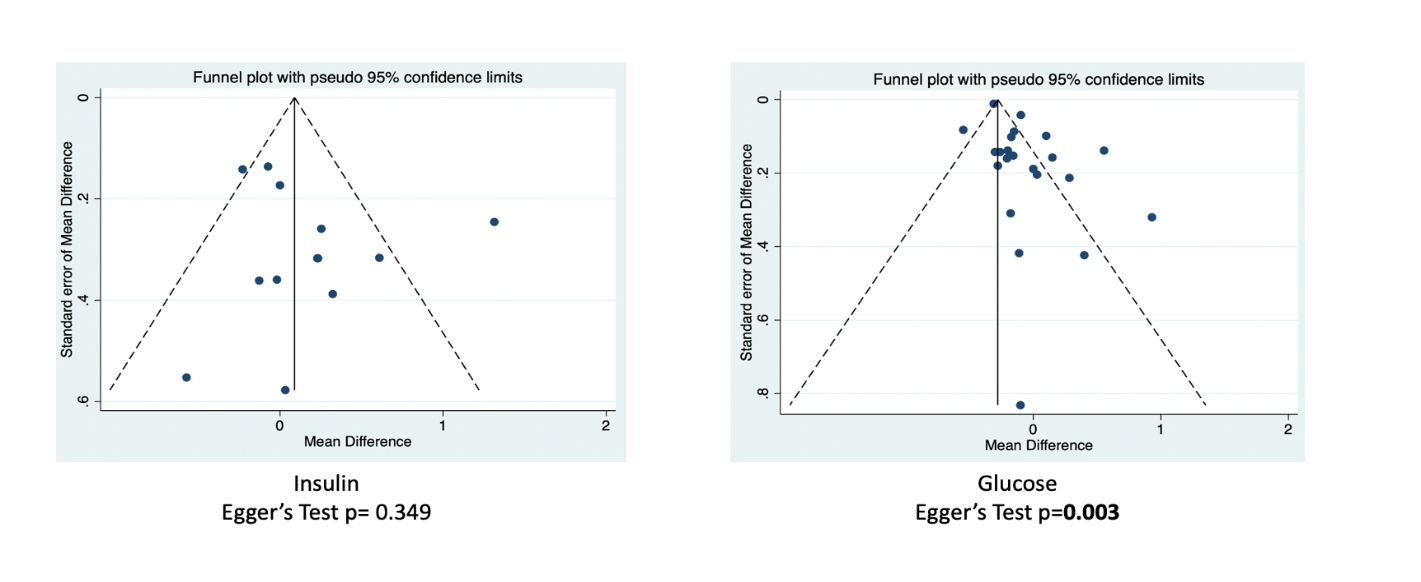


## CRP, c-reactive protein; LH, luteinizing hormone; FSH, follicle stimulating hormone.

## *Funnel plot visually illustrate the possibility of missing studies (publication bias) by plotting the standard error of mean difference (y axis) and the mean difference (x-axis). The dotted lines illustrate the area in which the studies (or expected studies) lies if there is no heterogeneity` and biases.

**Fig. S2 Leave-one-out analysis**

| Creatinine  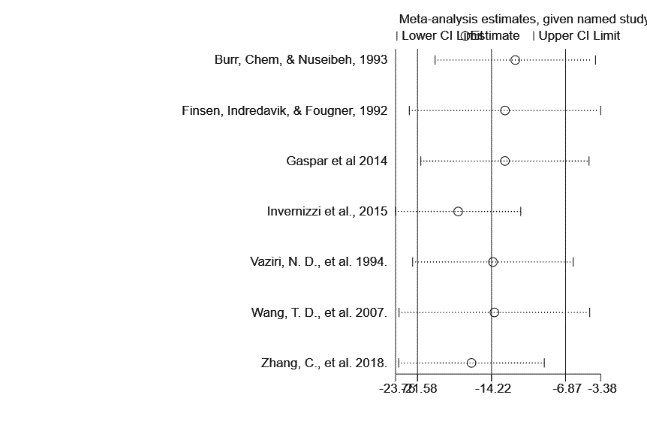 | CRP  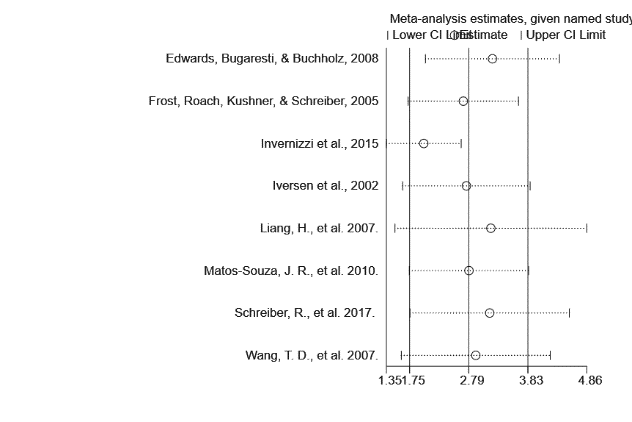 |
| --- | --- |
| FSH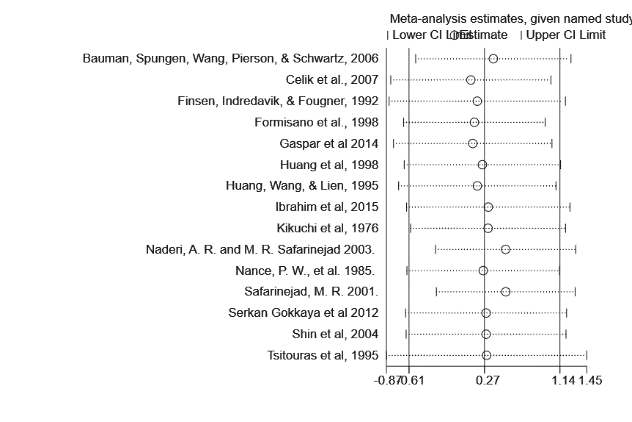 | LH  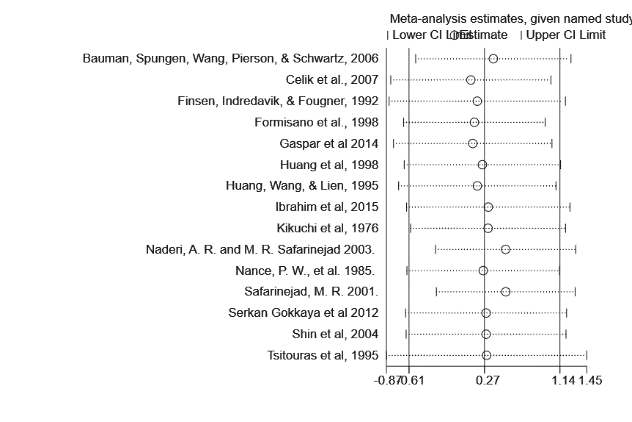 |
| Insulin  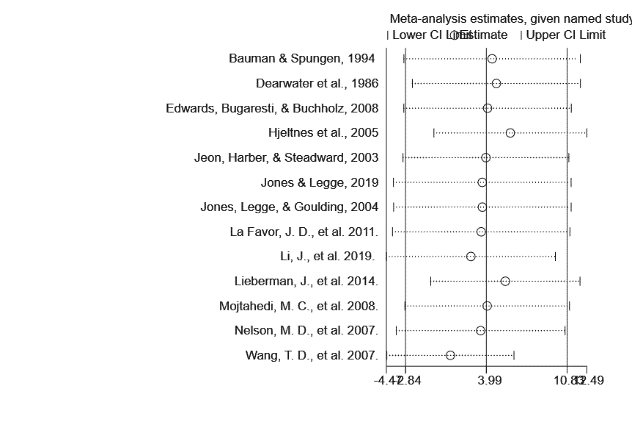 | Total testosterone  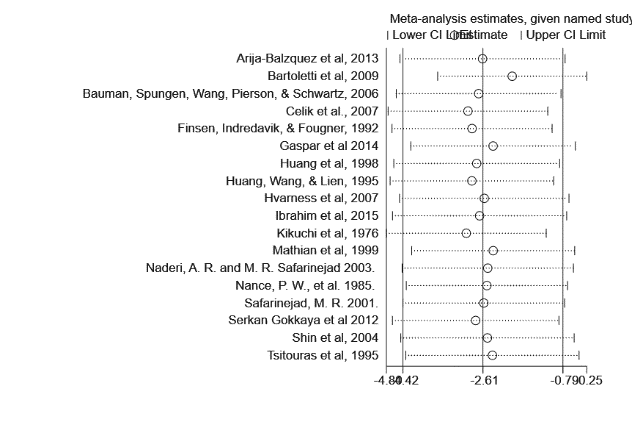 |
| Glucose   |  |

CRP, c-reactive protein; LH, luteinizing hormone; FSH, follicle stimulating hormone.

*Leave one out analysis recomputes the weighted mean difference upon removing one study, iteratively. This plot shows the studies removed (y-axis) and the recomputed mean difference/effect estimate (x-axis). Solid vertical lines represent the overall estimate/mean difference and the upper and lower limit of the complete analysis.

Each horizontal line represents the recomputed weighted mean difference upon removing the study indicated (circle as the effect estimate and bars as the confidence interval).

**Fig. S3 Bubble plot for FSH**

| FSH and age  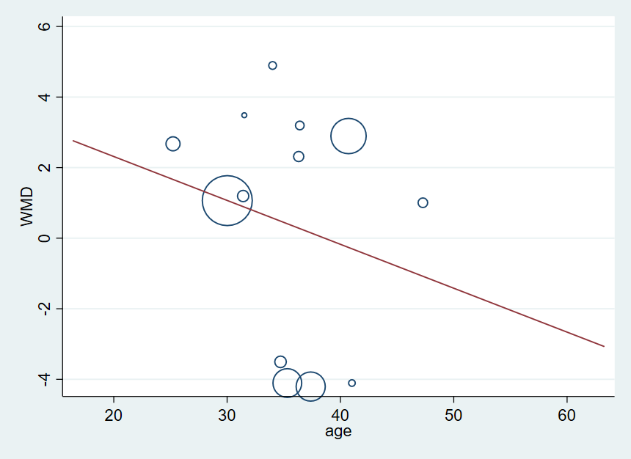 | FSH and injury duration  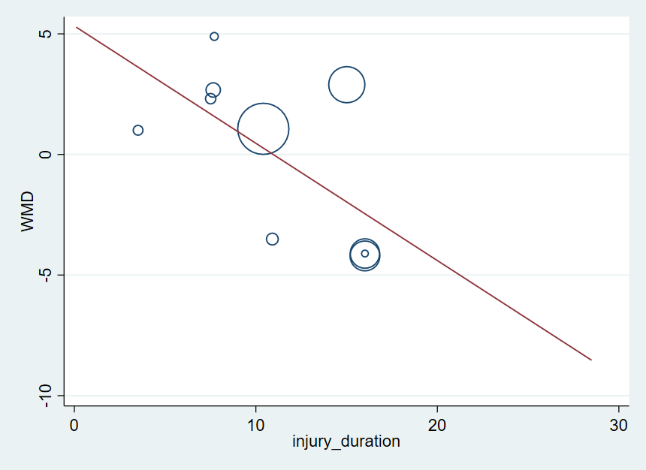 |
| --- | --- |
| FSH and sex (percent of males)  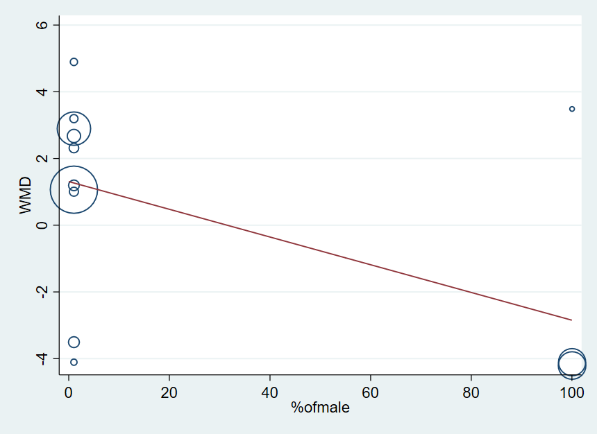 | FSH and number of participants  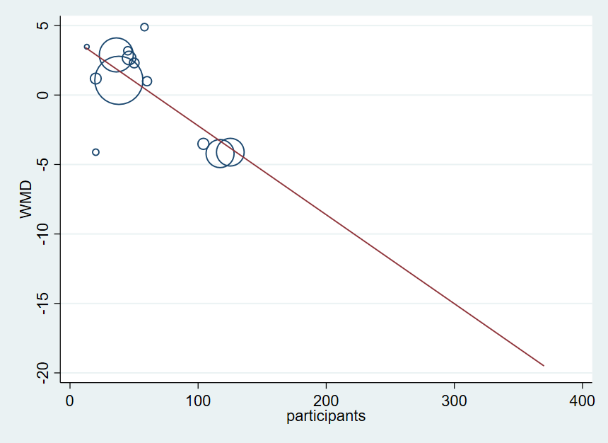 |

FSH, follicle stimulating hormone.

This shows the regression line with the weighted mean difference on y-axis and the predictor variable. The size points are the study estimates and the size of the points (bubble) is the weight given to the study. The beta coefficient of the regression can be seen in Table S8.

**Fig. S4 Bubble plot for LH**

| LH and age  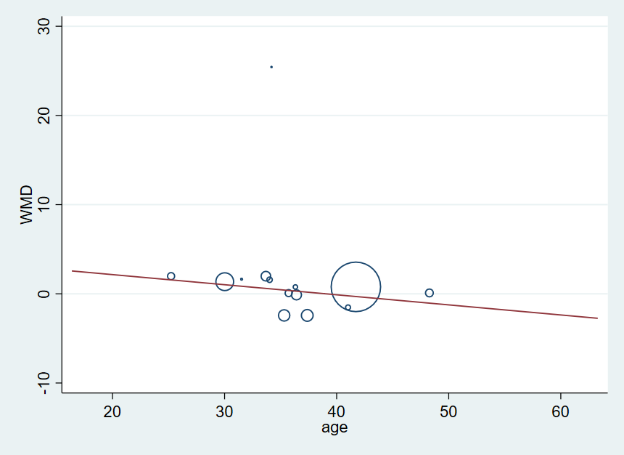 | LH and injury duration  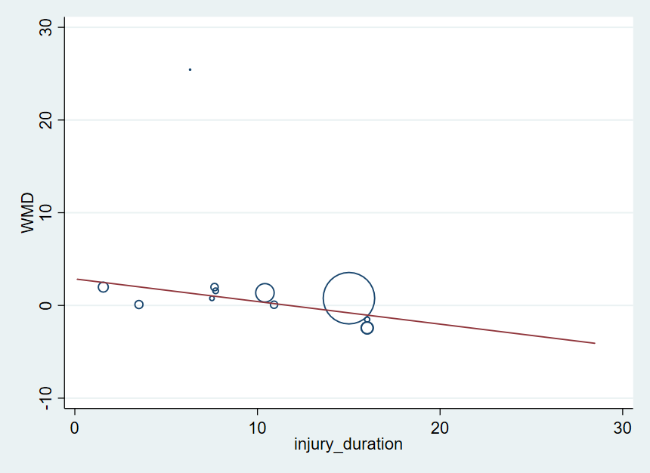 |
| --- | --- |
| LH and sex (percent of males)  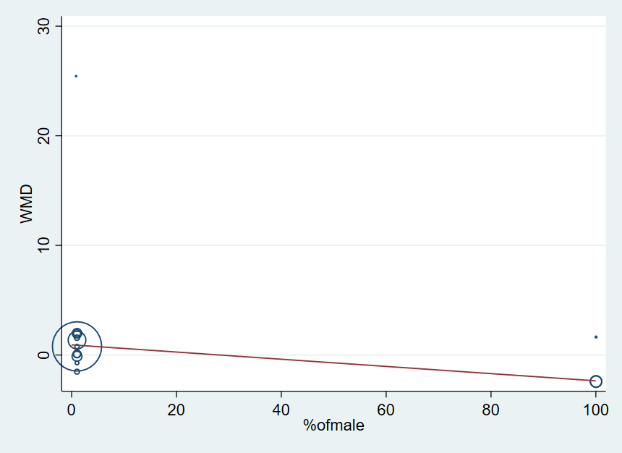 | LH and number of participants  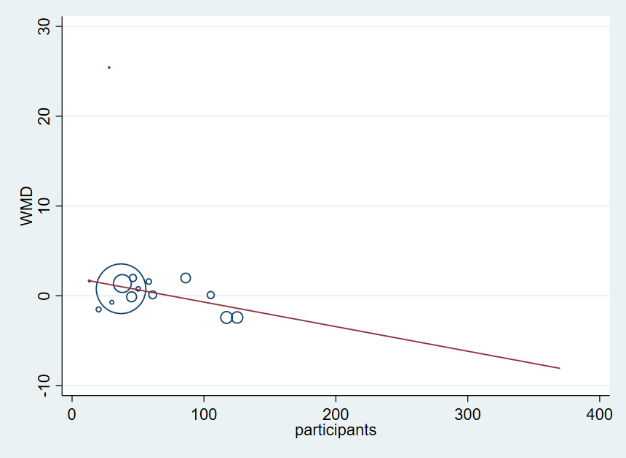 |

LH, luteinizing hormone.

This shows the regression line with the weighted mean difference on y-axis and the predictor variable. The size points are the study estimates and the size of the points (bubble) is the weight given to the study. The beta coefficient of the regression can be seen in Table S8.

**Fig. S5 Bubble plot for Insulin**

| Insulin and age  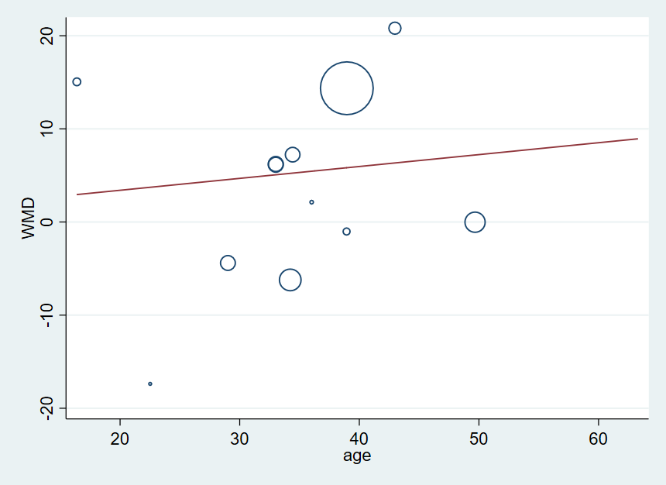 | Insulin and injury duration  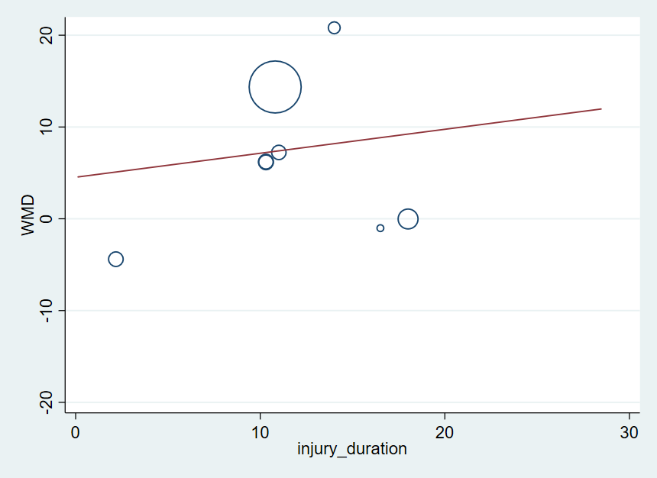 |
| --- | --- |
| Insulin and sex (percent of males)  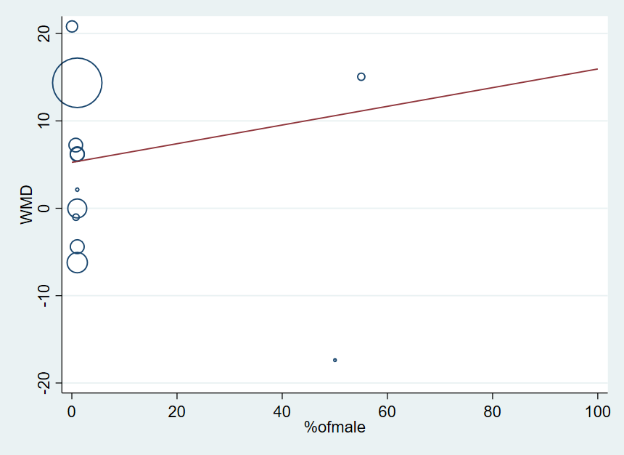 | Insulin and number of participants  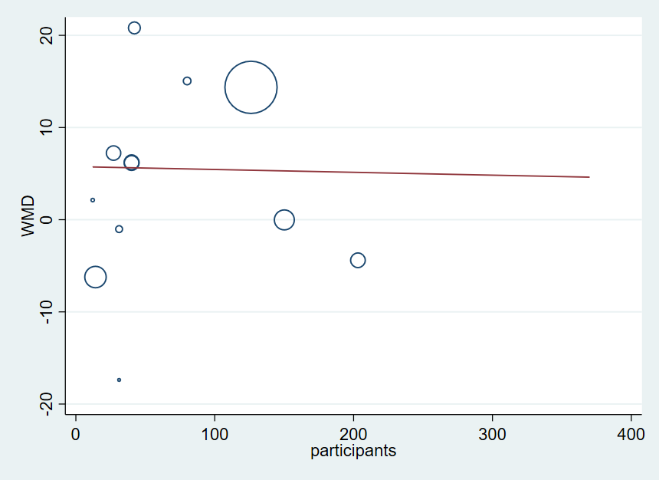 |

This shows the regression line with the weighted mean difference on y-axis and the predictor variable. The size points are the study estimates and the size of the points (bubble) is the weight given to the study.

**Fig. S6 Bubble plot for Total Testosterone**

| Insulin and age  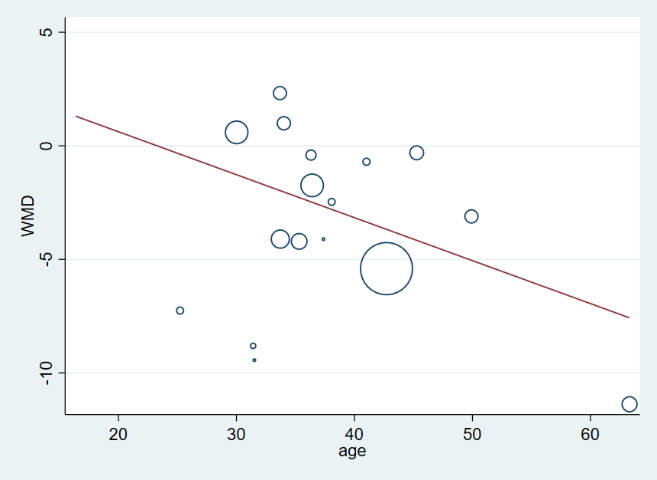 | Insulin and injury duration  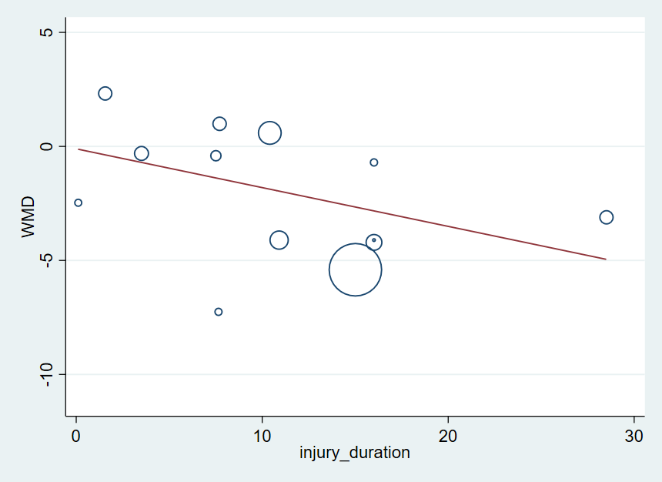 |
| --- | --- |
| Insulin and sex (percent of males)  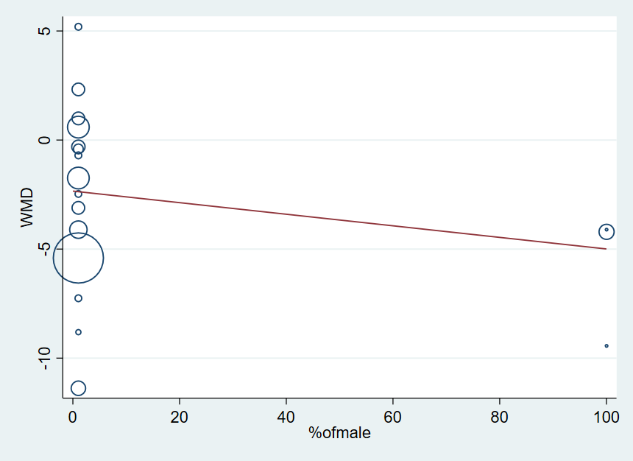 | Insulin and number of participants  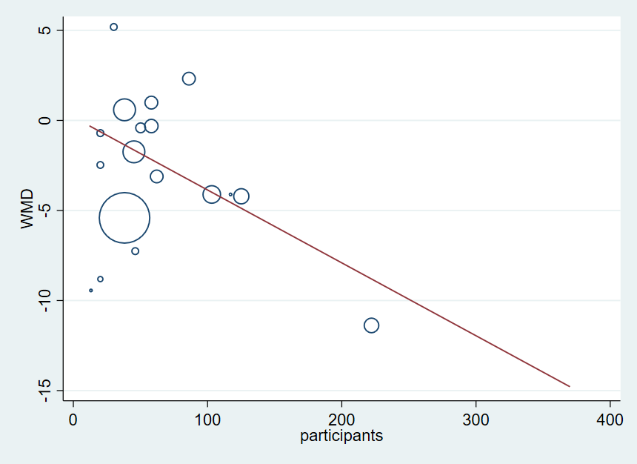 |

This shows the regression line with the weighted mean difference on y-axis and the predictor variable. The size points are the study estimates and the size of the points (bubble) is the weight given to the study. The beta coefficient of the regression can be seen in Table S8.

**Fig. S7 Bubble plot for Glucose**

| Glucose and age   | Glucose and injury duration   |
| --- | --- |
| Glucose and sex (percent of males)   | Glucose and number of participants   |

This shows the regression line with the weighted mean difference on y-axis and the predictor variable. The size points are the study estimates and the size of the points (bubble) is the weight given to the study.
